# Social and Physiological Context can Affect the Meaning of Physiological Synchrony

**DOI:** 10.1038/s41598-019-44667-5

**Published:** 2019-06-03

**Authors:** Chad Danyluck, Elizabeth Page-Gould

**Affiliations:** 1 Centers for American Indian and Alaska Native Health, Colorado School of Public Health, University of Colorado Denver Anschutz Medical Campus, Denver, USA; 2 Department of Psychology, University of Toronto, Toronto, Canada

**Keywords:** Autonomic nervous system, Human behaviour

## Abstract

Survival of many species, from insects and birds to human and non-human mammals, requires synchronized activity. Among humans, synchrony occurs even at the level of autonomic functioning; people interacting often show mutual, simultaneous changes in activity of the sympathetic or parasympathetic branches of the autonomic nervous system. Critically, autonomic reactivity predicts many mental states and, when synchronized, may reflect higher-order social processes like affiliation. Here, using data from 134 strangers interacting in pairs, we manipulated two features of social context to test their impact on synchrony in sympathetic and parasympathetic reactivity. Participants completed a knot-tying task within a collective reward (“cooperation”) or individual reward (“competition”) framework while conversing or not (“talking” condition). Autonomic reactivity varied by features of social context. Synchrony occurred across social contexts in both autonomic branches. We then examined how synchrony predicted affiliation. Sympathetic synchrony alone predicted affiliation yet social context and parasympathetic reactivity moderated associations between parasympathetic synchrony and affiliation. Thus, social and physiological context of parasympathetic synchrony predicted affiliation better than parasympathetic synchrony alone. We argue that social context and the degree of physiological reactivity underlying physiological synchrony, not the mere existence of physiological synchrony, are key to interpreting physiological synchrony as a social process.

## Introduction

Physiological synchrony seems “social,” because at least two organisms must be involved for the phenomena of physiological synchrony to be relevant^1–10^. There are reasons to pause on this assumption, however. At a basic level, it is unclear how much “social experience” is necessary for physiological synchrony to occur; physiological synchrony could simply reflect the mutually experienced demands of a shared environment. Given that the autonomic nervous system primarily serves to maintain homeostasis, the primary branches of the autonomic nervous system (i.e., the sympathetic and parasympathetic nervous systems) are only activated when an organism must *change* in order to continue maintaining homeostasis. Increases in sympathetic activity are associated with mental states like alertness, excitement, and subjective stress that, as a set, may relate to the novelty of the social context^11^. Comparatively, increased parasympathetic reactivity predicts relaxation and divided attention, which enables social engagement or work on complex tasks^12^. Decreased parasympathetic reactivity predicts focused attention^13,14^. In other words, psychological states are most closely related to *shifts* in physiological states from homeostatic baseline. Thus, we hypothesize that physiological synchrony is only socially meaningful in the context of physiological *reactivity*. That is, if branches of the autonomic nervous system are not activated, then we assume that physiological synchrony is more reflective of homeostatic processes in a shared environment. If branches of the autonomic nervous system are activated, then physiological synchrony is more likely to reflect higher-level, psychological processes.

Similarly, if physiological synchrony is related to social processes, it is unclear what social *function* it may serve. A naïve interpretation of synchrony would assume it is a prosocial process, whereby physiological connection implies mental connection. Among humans, physiological synchrony often co-occurs with social processes like affiliation^10^. Yet physiological synchrony during conflicts predicts relationship dissatisfaction^6^ and physiological synchrony even seems to be slightly stronger during social interactions between people who strongly dislike each other than it is among people who strongly like each other^15^. Relatedly, instances of discordant synchrony—when increased arousal in one person corresponds to decreased arousal in another—might have more to do with the structure of the interaction (for instance, taking turns) rather than with a strong social connection^16^.

The current research investigated how *much* and what *type* of social interaction is required for synchrony to occur. Human participants completed a simple motor task in the same room as each other for 5 minutes while autonomic nervous system activity was continuously monitored with electrocardiograph (ECG), with half the participants assigned to get to know each other (Talking Condition) or to not talk (No Talking Condition) during the task to manipulate how “social” the task was. After their interaction, participants completed self-report and behavioural measures of affiliation. The motor task required participants to use one hand to tie knots in a string anchored to their chairs. We also manipulated the type of social interaction by changing the reward structure of the task: Participants received points that made them eligible to win an Amazon gift card for each knot that their pair collectively tied (Cooperative Condition) or for each knot more that they tied relative to their partner (Competitive Condition). 134 strangers interacting in 67 same-sex, same-ethnicity pairs were randomly assigned to a 2 (Social Interaction, between-subjects: Talking, No Talking) × 2 (Interaction Orientation, between-subjects: Cooperative, Competitive) × 10 (Synchrony Over Time, within-subjects: 30-second epochs) mixed design.

From the resulting ECG data, we derived measures of sympathetic and parasympathetic reactivity. Using a mixed modeling approach, physiological synchrony was then statistically modeled by predicting one partner’s physiological measures from the physiological measures of the participant, while including the partner’s values from the previous time point to account for serial dependency in the data (all tests were two-tailed; see Methods). Physiological synchrony measures mutual changes in physiological reactivity over time. So long as changes in reactivity occur over time for both partners, physiological synchrony can be measured whether or not partners are highly reactive (i.e., partners can display low levels of physiological reactivity and still exhibit physiological synchrony if these small changes occur in unison).

## Sympathetic Activity

We examined synchrony of the sympathetic nervous system by modeling partner sympathetic reactivity as a function of the partner’s lagged sympathetic reactivity, participant’s sympathetic reactivity (“synchrony”), talking/no talking condition, cooperative/competitive condition, and all 2-way interactions and the 3-way interaction between those latter three variables in a 3-level multilevel model, where random intercepts were estimated for each pair and participant and a random slope for synchrony was estimated at the level of the pair. We used an autocorrelated synchrony matrix, and the degree of autocorrelation from minute-to-minute was estimated to be *ϕ* = 0.461, 95% CI [0.359, 0.552]. This model was determined to be ideal through a model comparison procedure detailed in Supplementary Information. The full model results are presented in Table 1. The results in Table 1 are divided into *fixed effects* and *random effects*. The random effects are the estimates of synchrony for each pair, and the fixed effects are the averages of the random effects.

**Table 1:** Model Predicting Sympathetic Synchrony.

Term	*Est*.	*CI*_*lower*_	*CI*_*upper*_	*SE*	*t-value*	*df*	*p-value*	*r*
Intercept	0.017	−0.105	0.139	0.063	0.268	1067	0.789	0.008
Lagged Partner	−0.263	−0.315	−0.210	0.027	−9.771	1067	<0.001	−0.287
Synchrony	0.061	−0.014	0.137	0.038	1.597	1067	0.111	0.049
Talking/No Talking	0.201	0.077	0.326	0.063	3.218	63	0.002	0.376
Cooperative/Competitive	0.138	0.014	0.262	0.063	2.207	63	0.031	0.268
Synchrony × Talking/No Talking	−0.050	−0.125	0.025	0.038	−1.308	1067	0.191	−0.040
Synchrony × Cooperative/Competitive	0.015	−0.060	0.090	0.038	0.380	1067	0.704	0.012
Talking/No Talking × Cooperative/Competitive	0.035	−0.089	0.160	0.063	0.567	63	0.573	0.071
Synchrony × Talking/No Talking × Cooperative/Competitive	0.036	−0.039	0.111	0.038	0.938	1067	0.349	0.029
**Random Effects**	***Variance Est*** *.*	***CI***_***lower***_	***CI***_***upper***_					
Dyad Intercept	0.075	0.018	0.306					
Synchrony	0.032	0.013	0.076					
Participant Intercept	0.199	0.103	0.385					
Residual	0.648	0.553	0.759					

*b* is the unstandardized slope, *CI*_*lower*_ and *CI*_*upper*_ are the lower and upper bounds of the slope’s 95% confidence interval, *SE* is the standard error of the slope, *df* are the degrees of freedom for that effect, *t-value* tests whether *b* is different from zero, *p-value* reflects the probability of *t-value* given the slope is zero, and *r* is a correlation coefficient reflecting the partial effect size. The social condition was coded with Talking = 1, No Talking = −1. The cooperative condition was coded with Cooperative = 1, Competitive = −1. Talking Cooperative (n = 32); Talking Competitive (n = 32); No Talking Cooperative (n = 30); No Talking Competitive (n = 40).

### Synchrony

**Figure 1:**
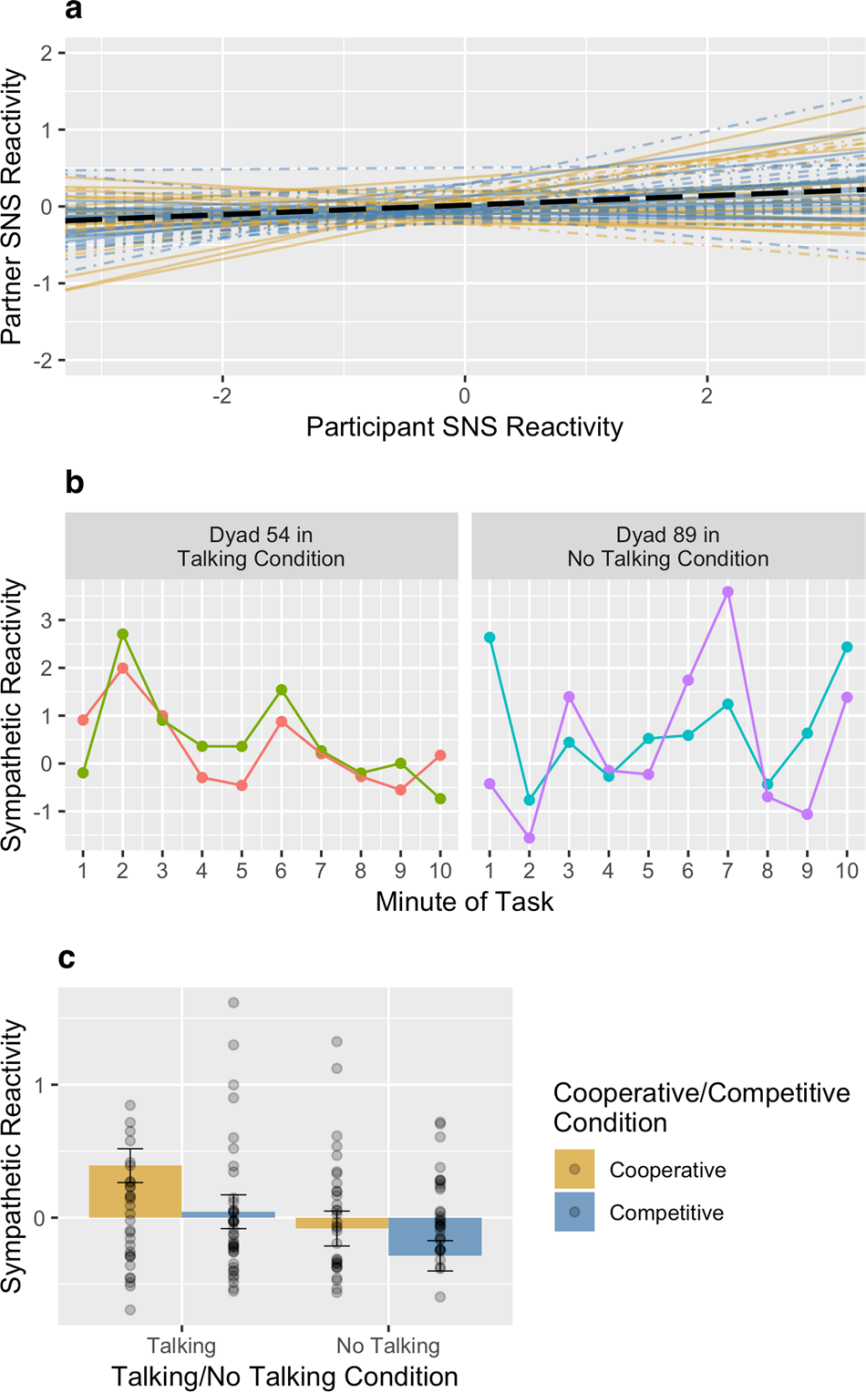
Reliability of the per-pair slope estimates for sympathetic synchrony (**a**). Sympathetic synchrony during the knot-tying task for two example pairs (**b**). Effect of experimental conditions on sympathetic reactivity (**c**). Error bars represent standard errors of the estimated marginal means.

The best demonstration of sympathetic synchrony is captured by the reliability of the per-pair slope estimates (random effects) for sympathetic synchrony (Fig. 1a), as this reflects the degree to which the per-pair synchrony estimates were reliable within pairs and reliably different between pairs. Indeed, the variance explained by sympathetic synchrony was reliably different from zero, ${\sigma }_{synchrony}^{2}$ = 0.032, 95% CI [0.013, 0.076], χ^2^(2) = 11.843, *p* = 0.003. To test if the sympathetic synchrony estimates were a result of the task or environment rather than the social pairing, we did an analysis where we randomly paired participants with other participants of the same sex who were assigned to the same experimental conditions and reran our model using the randomly-generated pairs. As further evidence that the synchrony estimates are meaningful, the sympathetic synchrony estimated among the random pairs was unreliable and no different from zero, ${\sigma }_{synchrony}^{2}$ = 0.005, χ^2^(2) = 0.323, *p* = 0.851. Figure 1b demonstrates synchrony by showing sympathetic reactivity over the course of the knot-tying task for two example pairs, one in the talking condition (left panel) and the other in the no talking condition (right panel).

Across pairs, sympathetic synchrony was not reliably concordant (positively related) or discordant (negatively related), although synchrony trended towards being concordant on average, *b* = 0.061, *SE* = 0.038, 95% CI [−0.014, 0.137], *t*(1067) = 1.597, *p* = 0.111, *r* = 0.049, reflecting that covariation of sympathetic reactions tended to go in the same direction but some pairs were synchronous but in opposing directions (see Fig. 1a). Sympathetic synchrony was not moderated by experimental condition, *b* = 0.036, *SE* = 0.038, 95% CI [−0.039, 0.111], *t*(1067) = 0.938, *p* = 0.349, *r* = 0.029.

### Reactivity

There were main effects on sympathetic reactivity for both experimental conditions. Participants in the talking condition were more sympathetically activated than participants who were instructed not to talk, *b* = 0.201, *SE* = 0.077, 95% CI [0.077, 0.326], *t*(63) = 3.218, *p* = 0.002, *r* = 0.376. Somewhat surprisingly, there was greater sympathetic activation when participants were cooperating with each other than when they were competing, *b* = 0.138, *SE* = 0.063, 95% CI [0.014, 0.262], *t*(63) = 2.207, *p* = 0.031, *r* = 0.268, albeit, this effect was less strong than the effect in the talking condition. There was no interaction between conditions, *b* = 0.035, *SE* = 0.063, 95% CI [−0.089, 0.160], *t*(63) = 0.567, *p* = 0.573, *r* = 0.071.

Additionally, we tested for significant increases in sympathetic activity from baseline in each condition (i.e., sympathetic reactivity). People displayed significant sympathetic reactivity in the talking cooperative condition, *M* = 0.391, *SE* = 0.127, 95% CI [0.137, 0.645]. These effects are depicted in Fig. 1c and are reported in full in Supplementary Table S1.

## Parasympathetic Activity

We examined synchrony of the parasympathetic nervous system by modeling partner parasympathetic reactivity as a function of the partner’s lagged parasympathetic reactivity, participant’s parasympathetic reactivity (“synchrony”), talking/no talking condition, cooperative/competitive condition, and all 2-way interactions and the 3-way interaction between those latter three variables in a 3-level multilevel model, where random intercepts were estimated for each pair and participant and a random slope for synchrony and the partner’s lagged parasympathetic activity was estimated at the level of the pair. We used an autocorrelated covariance matrix, and the degree of autocorrelation from minute-to-minute was estimated to be *ϕ* = 0.519, 95% CI [0.403, 0.618]. This model was determined to be ideal through a model comparison procedure detailed in the supplemental materials. The full model results are presented in Table 2. As with Table 1, the random effects show the degree of synchrony within each pair and the fixed effects represent averages of the random effects.

**Table 2:** Model Predicting Parasympathetic Synchrony.

Term	*Est*.	*CI*_*lower*_	*CI*_*upper*_	*SE*	*t-value*	*df*	*p-value*	*r*
Intercept	0.010	−0.118	0.139	0.066	0.156	1067	0.876	0.005
Lagged Partner	−0.267	−0.328	−0.205	0.031	−8.510	1067	<0.001	−0.252
Synchrony	0.094	0.024	0.164	0.036	2.613	1067	0.009	0.08
Talking/No Talking	0.175	0.044	0.305	0.066	2.664	63	0.010	0.318
Cooperative/Competitive	−0.064	−0.194	0.067	0.066	−0.969	63	0.336	−0.121
Synchrony × Talking/No Talking	−0.011	−0.080	0.057	0.035	−0.321	1067	0.749	−0.010
Synchrony × Cooperative/Competitive	0.001	−0.068	0.069	0.035	0.025	1067	0.980	0.001
Talking/No Talking × Cooperative/Competitive	−0.096	−0.227	0.034	0.066	−1.470	63	0.147	−0.182
Synchrony × Talking/No Talking × Cooperative/Competitive	0.047	−0.021	0.116	0.035	1.349	1067	0.178	0.041
**Random Effects**	***Variance Est*** *.*	***CI***_***lower***_	***CI***_***upper***_					
Dyad Intercept	0.005	0.000	5.043					
Synchrony	0.014	0.004	0.053					
Participant Intercept	0.434	0.311	0.606					
Residual	0.436	0.354	0.535					

*b* is the unstandardized slope, *CI*_*lower*_ and *CI*_*upper*_ are the lower and upper bounds of the slope’s 95% confidence interval, *SE* is the standard error of the slope, *df* are the degrees of freedom for that effect, *t-value* tests whether *b* is different from zero, *p-value* reflects the probability of *t-value* given the slope is zero, and *r* is a correlation coefficient reflecting the partial effect size. The social condition was coded with Talking = 1, No Talking = −1. The cooperative condition was coded with Cooperative = 1, Competitive = −1. Talking Cooperative (n = 32); Talking Competitive (n = 32); No Talking Cooperative (n = 30); No Talking Competitive (n = 40).

### Synchrony

**Figure 2:**
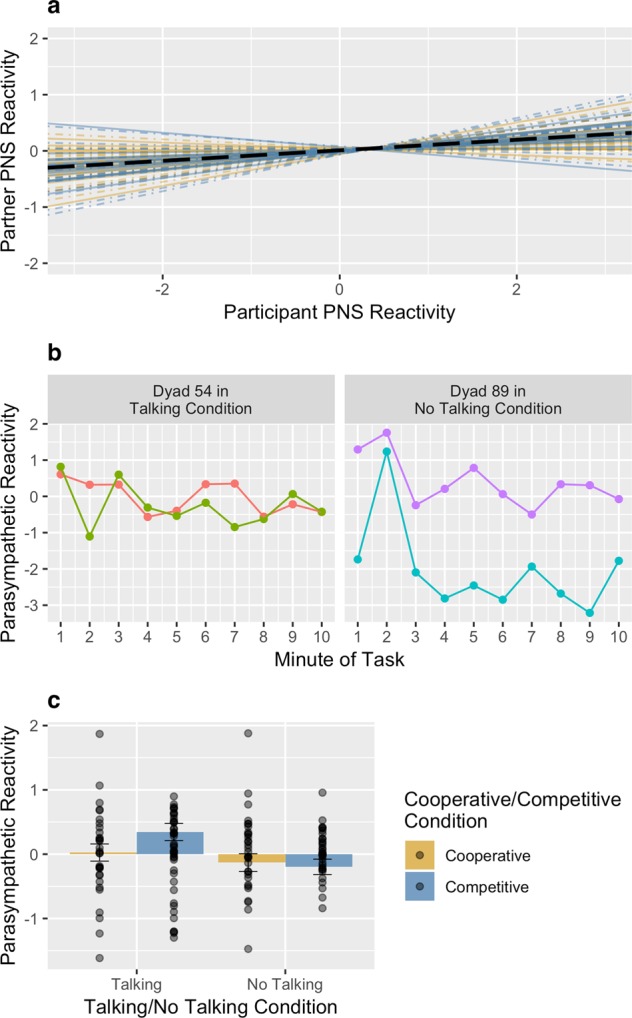
Reliability of the per-pair slope estimates for parasympathetic synchrony (**a**). Parasympathetic synchrony during the knot-tying task for two example pairs (**b**). Effect of experimental conditions on parasympathetic reactivity (**c**). Error bars represent standard errors of the estimated marginal means.

The random slopes for parasympathetic synchrony suggested that parasympathetic synchrony was reliable within pairs and that there was a reliable difference between pairs, ${\sigma }_{synchrony}^{2}$ = 0.024, 95% CI [0.009, 0.069], χ^2^(3) = 9.655, *p* = 0.022. Figure 2a plots parasympathetic synchrony effects for each pair. Parasympathetic synchrony estimated among random pairs was no different from zero, ${\sigma }_{synchrony}^{2}$ = 0.006, χ^2^(3) = 1.61, *p* = 0.657, providing further evidence that parasympathetic synchrony was a process that occurred between the two partners and not simply due to the task itself. Figure 2b demonstrates parasympathetic synchrony by showing parasympathetic reactivity during the knot-tying task in the same example pairs as were shown for Fig. 1b.

Across all pairs, there was an average, positive effect of parasympathetic synchrony, *b* = 0.094, *SE* = 0.036, 95% CI [0.024, 0.164], *t*(1067) = 2.613, *p* = 0.009, *r* = 0.08. As was seen with sympathetic synchrony, parasympathetic synchrony occurred across all experimental conditions (i.e., it was unmoderated by experimental conditions), *b* = 0.047, *SE* = 0.035, 95% CI [−0.021, 0.116], *t*(1067) = 1.349, *p* = 0.178, *r* = 0.041.

### Reactivity

There was only a difference in parasympathetic reactivity between the talking/no talking experimental conditions, such that participants in the talking condition showed greater parasympathetic activation than participants in the no talking condition, *b* = 0.175, *SE* = 0.066, 95% CI [0.044, 0.305], *t*(63) = 2.664, *p* = 0.010, *r* = 0.318. There was no difference in parasympathetic activation as a function of cooperation or competition, *b* = −0.064, *SE* = 0.066, 95% CI [−0.194, 0.067], *t*(63) = −0.969, *p* = 0.336, *r* = −0.121, nor did the cooperation/competition manipulation moderate the differences between the talking and no talking conditions, *b* = −0.096, *SE* = 0.066, 95% CI [−0.227, 0.034], *t*(63) = −1.469, *p* = 0.147, *r* = −0.182.

Additionally, we tested for significant differences in parasympathetic activity from baseline in each condition (i.e., parasympathetic reactivity). People displayed significant parasympathetic reactivity in the talking competitive condition, *M* = 0.345, *SE* = 0.134, 95% CI [0.078, 0.612]. These effects are depicted in Fig. 2c and are reported in full in Supplementary Table S1.

## Synchrony and Social Processes

Our more general interest in physiological synchrony is how synchrony during social interactions relates to social processes that ultimately lead to friendship. Thus, we conducted exploratory analyses on the relation between physiological synchrony and reactivity with measures of affiliation that we have previously examined in relation to physiological synchrony^10^, specifically perceived similarity, friendship interest, positive and negative affect, and desire to exchange contact information. The interaction term combined physiological reactivity and coefficients for the random slopes of synchrony that were extracted from our preliminary analyses of physiological synchrony. The bivariate correlations between sympathetic and parasympathetic synchrony and reactivity and measures of affiliation are provided in Supplementary Table S2.

As many of these measures of affiliation were correlated, we conducted our analyses using multivariate multilevel models that estimated the effects of sympathetic and parasympathetic synchrony and reactivity for each dependent variable while accounting for the covariance between dependent variables. Unfortunately, though, the Hessian matrix for our multivariate models were not positive-definite with positive affect and email exchange included, so we had to remove these social outcomes from our analyses. Ultimately, we modeled perceived similarity, friendship interest, and negative affect simultaneously using two multivariate multilevel models, where one model examined effects of sympathetic synchrony and reactivity and their interactions with the experimental conditions and the other model examined the effects of parasympathetic synchrony and reactivity and their interactions with the experimental conditions. The random effects for these models included random intercepts for each dependent variable, which were allowed to be correlated with each other. These random effects were nested within each pair. In addition, to implement the multivariate multilevel models, the residuals were allowed to be different for each dependent variable. Full model results are presented below separately for each dependent variable, but they were estimated together in the same model.

## Perceived Similarity

### Sympathetic nervous system

**Figure 3:**
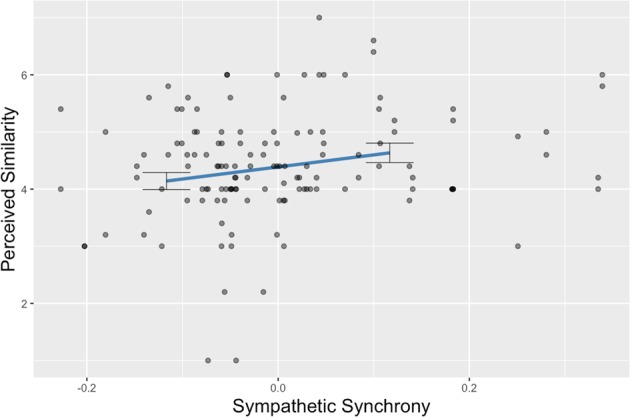
Association between sympathetic synchrony and perceived similarity. Error bars represent standard errors of the estimated marginal means.

The full results for the sympathetic nervous system and perceived similarity are presented in Table 3. There were main effects of both sympathetic synchrony and reactivity for perceived similarity (for an elaboration on the latter effect, see Supplementary Information). Across conditions, sympathetic synchrony predicted greater perceptions of similarity with one’s partner, *b* = 2.061, *SE* = 1.031, 95% CI [0.157, 3.964], *t*(288) = 2.00, *p* = 0.046, *r* = 0.117, albeit, with a small effect (Fig. 3). Moreover, sympathetic reactivity did not moderate the association between sympathetic synchrony and perceived similarity, thus, sympathetic synchrony predicted perceived similarity regardless of the social and physiological context.

**Table 3:** Multivariate Model Predicting Perceived Similarity and Friendship Interest from Sympathetic Synchrony and Reactivity.

Term	Perceived Similarity							
	*b*	*CI*_*lower*_	*CI*_*upper*_	*SE*	*t-value*	*df*	*p-value*	*r*
Intercept	4.400	4.206	4.593	0.105	41.976	288	<0.001	0.927
Talking/No Talking	0.103	−0.090	0.297	0.105	0.987	288	0.325	0.058
Cooperative/Competitive	0.099	−0.094	0.293	0.105	0.947	288	0.345	0.056
Reactivity	0.479	0.153	0.805	0.177	2.716	288	0.007	0.158
Synchrony	2.061	0.157	3.964	1.031	2.000	288	0.046	0.117
Talking/No Talking × Cooperative/Competitive	0.087	−0.107	0.281	0.105	0.830	288	0.407	0.049
Talking/No Talking × Reactivity	0.457	0.131	0.783	0.177	2.591	288	0.010	0.151
Cooperative/Competitive × Reactivity	−0.364	−0.690	−0.038	0.177	−2.062	288	0.040	−0.121
Talking/No Talking × Synchrony	−1.079	−2.982	0.825	1.031	−1.047	288	0.296	−0.062
Cooperative/Competitive × Synchrony	−0.071	−1.974	1.833	1.031	−0.069	288	0.945	−0.004
Reactivity × Synchrony	−1.873	−5.526	1.779	1.978	−0.947	288	0.344	−0.056
Talking/No Talking × Cooperative/Competitive × Reactivity	−0.289	−0.615	0.037	0.177	−1.639	288	0.102	−0.096
Talking/No Talking × Cooperative/Competitive × Synchrony	−0.456	−2.360	1.447	1.031	−0.443	288	0.658	−0.026
Talking/No Talking × Reactivity × Synchrony	−1.527	−5.180	2.126	1.978	−0.772	288	0.441	−0.045
Cooperative/Competitive × Reactivity × Synchrony	0.412	−3.241	4.065	1.978	0.208	288	0.835	0.012
Talking/No Talking × Cooperative/Competitive × Reactivity × Synchrony	−0.659	−4.312	2.994	1.978	−0.333	288	0.739	−0.020
**Term**	**Friendship Interest**							
	***b***	***CI***_***lower***_	***CI***_***upper***_	***SE***	***t-value***	***df***	***p-value***	***r***
Intercept	4.840	4.621	5.059	0.118	40.890	288	<0.001	0.924
Talking/No Talking	0.229	0.011	0.448	0.118	1.936	288	0.054	0.113
Cooperative/Competitive	0.222	0.003	0.441	0.118	1.876	288	0.062	0.110
Reactivity	0.694	0.343	1.046	0.190	3.650	288	<0.001	0.210
Synchrony	0.248	−1.901	2.398	1.164	0.213	288	0.831	0.013
Talking/No Talking × Cooperative/Competitive	0.076	−0.142	0.295	0.118	0.645	288	0.519	0.038
Talking/No Talking × Reactivity	0.603	0.251	0.954	0.190	3.167	288	0.002	0.183
Cooperative/Competitive × Reactivity	−0.720	−1.071	−0.368	0.190	−3.784	288	<0.001	−0.218
Talking/No Talking × Synchrony	−0.216	−2.366	1.933	1.164	−0.186	288	0.853	−0.011
Cooperative/Competitive × Synchrony	0.212	−1.938	2.362	1.164	0.182	288	0.855	0.011
Reactivity × Synchrony	−1.455	−5.426	2.516	2.150	−0.677	288	0.499	−0.040
Talking/No Talking × Cooperative/Competitive × Reactivity	−0.498	−0.849	−0.146	0.190	−2.617	288	0.009	−0.152
Talking/No Talking × Cooperative/Competitive × Synchrony	0.003	−2.147	2.153	1.164	0.003	288	0.998	0.000
Talking/No Talking × Reactivity × Synchrony	−2.306	−6.277	1.665	2.150	−1.073	288	0.284	−0.063
Cooperative/Competitive × Reactivity × Synchrony	0.915	−3.056	4.886	2.150	0.425	288	0.671	0.025
Talking/No Talking × Cooperative/Competitive × Reactivity × Synchrony	0.577	−3.394	4.548	2.150	0.268	288	0.789	0.016

*b* is the unstandardized slope, *CI*_*lower*_ and *CI*_*upper*_ are the lower and upper bounds of the slope’s 95% confidence interval, *SE* is the standard error of the slope, *df* are the degrees of freedom for that effect, *t-value* tests whether *b* is different from zero, *p-value* reflects the probability of *t-value* given the slope is zero, and *r* is a correlation coefficient reflecting the partial effect size. The talking condition was coded with Talking = 1, No Talking = −1. The cooperative condition was coded with Cooperative = 1, Competitive = −1. Talking Cooperative (*n* = 32); Talking Competitive (*n* = 32); No Talking Cooperative (*n* = 30); No Talking Competitive (*n* = 40).

### Parasympathetic nervous system

The full results for the parasympathetic nervous system and perceived similarity are presented in Table 4. As can be seen in that table, only parasympathetic reactivity was related to perceived similarity, and those results are reported in detail in Supplementary Information.

**Table 4:** Multivariate Model Predicting Perceived Similarity and Friendship Interest from Parasympathetic Synchrony and Reactivity.

Term	Perceived Similarity							
	*b*	*CI*_*lower*_	*CI*_*upper*_	*SE*	*t-value*	*df*	*p-value*	*r*
Intercept	4.346	4.155	4.538	0.104	41.884	288	<0.001	0.927
Talking/No Talking	0.099	−0.092	0.291	0.104	0.959	288	0.338	0.056
Cooperative/Competitive	0.190	−0.002	0.382	0.104	1.832	288	0.068	0.107
Reactivity	−0.004	−0.243	0.235	0.129	−0.027	288	0.978	−0.002
Synchrony	−0.884	−2.912	1.144	1.098	−0.805	288	0.422	−0.047
Talking/No Talking × Cooperative/Competitive	0.126	−0.066	0.317	0.104	1.212	288	0.227	0.071
Talking/No Talking × Reactivity	0.245	0.006	0.484	0.129	1.891	288	0.060	0.111
Cooperative/Competitive × Reactivity	−0.023	−0.262	0.216	0.129	−0.177	288	0.859	−0.010
Talking/No Talking × Synchrony	−1.020	−3.048	1.008	1.098	−0.929	288	0.354	−0.055
Cooperative/Competitive × Synchrony	−0.582	−2.610	1.446	1.098	−0.530	288	0.596	−0.031
Reactivity × Synchrony	2.213	−0.492	4.917	1.464	1.511	288	0.132	0.089
Talking/No Talking × Cooperative/Competitive × Reactivity	−0.429	−0.668	−0.190	0.129	−3.317	288	0.001	−0.192
Talking/No Talking × Cooperative/Competitive × Synchrony	0.966	−1.062	2.994	1.098	0.880	288	0.380	0.052
Talking/No Talking × Reactivity × Synchrony	2.460	−0.245	5.164	1.464	1.680	288	0.094	0.098
Cooperative/Competitive × Reactivity × Synchrony	−2.032	−4.737	0.672	1.464	−1.388	288	0.166	−0.082
Talking/No Talking × Cooperative/Competitive × Reactivity × Synchrony	−0.238	−2.942	2.467	1.464	−0.162	288	0.871	−0.010
**Term**	**Friendship Interest**							
	***b***	***CI***_***lower***_	***CI***_***upper***_	***SE***	***t-value***	***df***	***p-value***	***r***
Intercept	4.778	4.569	4.987	0.113	42.245	288	<0.001	0.928
Talking/No Talking	0.238	0.029	0.447	0.113	2.104	288	0.036	0.123
Cooperative/Competitive	0.284	0.076	0.493	0.113	2.515	288	0.012	0.147
Reactivity	0.109	−0.137	0.355	0.133	0.818	288	0.414	0.048
Synchrony	−0.694	−2.903	1.515	1.196	−0.581	288	0.562	−0.034
Talking/No Talking × Cooperative/Competitive	0.100	−0.109	0.309	0.113	0.885	288	0.377	0.052
Talking/No Talking × Reactivity	0.301	0.055	0.547	0.133	2.261	288	0.025	0.132
Cooperative/Competitive × Reactivity	−0.087	−0.332	0.159	0.133	−0.650	288	0.516	−0.038
Talking/No Talking × Synchrony	−0.229	−2.438	1.980	1.196	−0.191	288	0.849	−0.011
Cooperative/Competitive × Synchrony	1.545	−0.664	3.754	1.196	1.292	288	0.198	0.076
Reactivity × Synchrony	3.231	0.466	5.997	1.497	2.158	288	0.032	0.126
Talking/No Talking × Cooperative/Competitive × Reactivity	−0.402	−0.648	−0.156	0.133	−3.020	288	0.003	−0.175
Talking/No Talking × Cooperative/Competitive × Synchrony	1.773	−0.436	3.982	1.196	1.482	288	0.139	0.087
Talking/No Talking × Reactivity × Synchrony	4.227	1.461	6.992	1.497	2.823	288	0.005	0.164
Cooperative/Competitive × Reactivity × Synchrony	−4.306	−7.072	−1.541	1.497	−2.876	288	0.004	−0.167
Talking/No Talking × Cooperative/Competitive × Reactivity × Synchrony	−1.944	−4.710	0.821	1.497	−1.298	288	0.195	−0.076

*b* is the unstandardized slope, *CI*_*lower*_ and *CI*_*upper*_ are the lower and upper bounds of the slope’s 95% confidence interval, *SE* is the standard error of the slope, *df* are the degrees of freedom for that effect, *t-value* tests whether *b* is different from zero, *p-value* reflects the probability of *t-value* given the slope is zero, and *r* is a correlation coefficient reflecting the partial effect size. The talking condition was coded with Talking = 1, No Talking = −1. The cooperative condition was coded with Cooperative = 1, Competitive = −1. Talking Cooperative (*n* = 32); Talking Competitive (*n* = 32); No Talking Cooperative (*n* = 30); No Talking Competitive (*n* = 40).

## Friendship Interest

### Sympathetic nervous system

The full results for the sympathetic nervous system and friendship interest are presented in Table 3. In the sympathetic nervous system, reactivity was primarily related to friendship interest, and those results are reported in full in Supplementary Information.

### Parasympathetic nervous system

**Figure 4:**
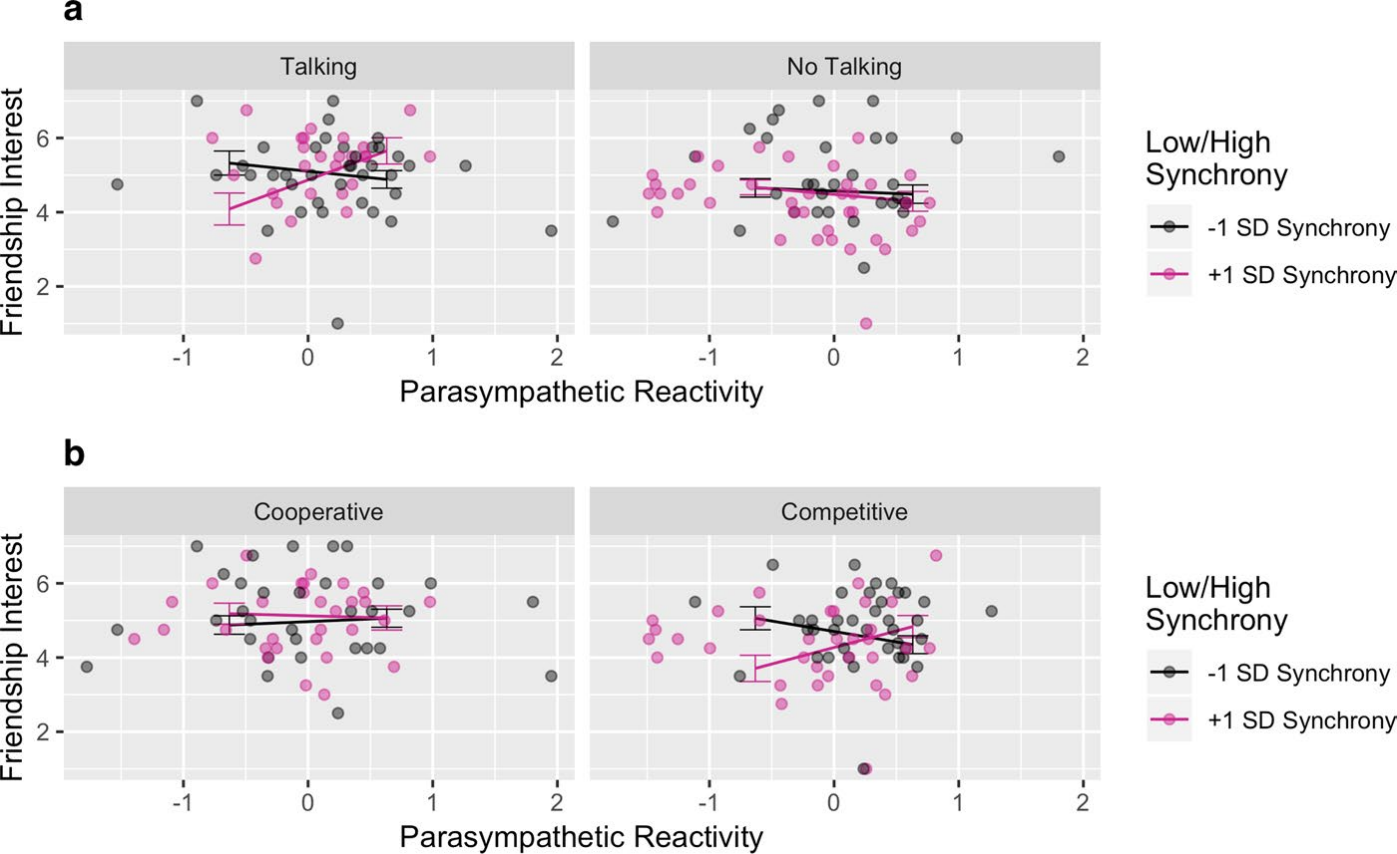
Moderation of the relationship between parasympathetic synchrony and reactivity and friendship interest by no talking/talking conditions (**a**). Moderation of relationship between parasympathetic synchrony and reactivity and friendship interest by competition/cooperation conditions (**b**).

The full results for the parasympathetic nervous system and friendship interest are presented in Table 4. Friendship interest was predicted by a 3-way interaction between the talking conditions, parasympathetic reactivity, and parasympathetic synchrony, *b* = 4.227, *SE* = 1.497, 95% CI [1.461, 6.992], *t*(288) = 2.823, *p* = 0.005, *r* = 0.164. After adjusting *p*-values for multiple comparisons, the key difference was that talking appeared to drive friendship interest when participants were both parasympathetically activated (i.e., both parasympathetic reactivity and synchrony were high), *adj. p* = 0.048 (Fig. 4a).

There was also a 3-way interaction between the cooperative/competitive conditions, parasympathetic reactivity, and parasympathetic synchrony, *b* = −4.306, *SE* = 1.497, 95% CI [−7.072, −1.541], *t*(288) = −2.876, *p* = 0.004, *r* = −0.167. As shown in Fig. 4b, partners who both showed decreases in parasympathetic activity (i.e., decreased parasympathetic reactivity and high parasympathetic synchrony) were more interest in friendship with each other when they were cooperating instead of competing, *adj. p* = 0.027.

Further results for parasympathetic reactivity predicting friendship interest are provided in Supplementary Information.

## Negative Affect

Neither sympathetic nor parasympathetic reactivity or synchrony predicted negative affect. Full results are presented in Supplementary Table S6.

Consistent with prior work^7,8^, physiological synchrony was observed in both branches of the autonomic nervous system and across social contexts. Yet different social contexts caused different levels of physiological reactivity. People who talked while cooperating typically displayed mutual increases in sympathetic reactivity, which has been related to task engagement in past work^11^. People who talked while competing generally exhibited mutual increases in parasympathetic reactivity, which has been related to social processing^12^. In the no talking condition, on average, people who competed displayed mutual decreases in sympathetic reactivity. In all other conditions, partners typically displayed a mutual relative lack of change in sympathetic and parasympathetic activity. Thus, even though physiological synchrony was ubiquitous, the *physiological context* of physiological synchrony between partners varied depending on what social context people shared.

In every condition, strangers quickly went “in sync” and did so in each branch of the autonomic nervous system whether they were high or low in arousal. Whether the social or physiological context of synchrony contributed to social outcomes, however, depended on which branch of the autonomic nervous system displayed synchrony. Sympathetic synchrony predicted perceived similarity between partners and this was true *regardless of the social or physiological context*. That is, sharing similar amounts sympathetic reactivity was sufficient to increase perceptions of similarity regardless of social context and no matter the arousal levels partners shared. Although this finding runs counter to the expectation that physiological synchrony would be most socially meaningful in the context of physiological reactivity, the finding is supported by prior work demonstrating a link between sympathetic synchrony and affiliation among strangers in similarly benign settings^10^.

One possible explanation for this finding is that patterns of sympathetic arousal may correlate with observable body movements (and by extension a lack of arousal may correlate with a lack of body movement) that might predict perceived similarity if shared among partners. Indeed, prior research has demonstrated synchrony of body movements in married couples, in conjunction with sympathetic synchrony, and found synchrony in this set of measures to be related to individual differences in marital satisfaction^6^. Moreover, behavioural synchrony predicts measures of affiliation, including perceived similarity^17^. The present study does not examine whether sympathetic activity corresponds to body movements but it seems plausible that such an association might explain why sympathetic synchrony predicts perceived similarity, regardless of partners’ shared arousal levels.

By comparison, variation in both social and physiological context turned out to be a crucial moderator of the association between parasympathetic synchrony and friendship interest. People for whom parasympathetic synchrony and parasympathetic reactivity was high generally reported more friendship interest when the social context permitted conversation than when it did not. In other words, when parasympathetic activity increased during a social interaction, parasympathetic synchrony only mattered for the development of friendship between strangers who could converse. When people exhibited decreases in parasympathetic reactivity, parasympathetic synchrony predicted more friendship interest during cooperation than during competition. Thus, synchronized decreases in parasympathetic activity contribute to friendship interest when strangers are cooperating but not when competing.

Increases in parasympathetic activity predicts relaxation and divided attention, which enables social engagement or work on complex tasks, whereas decreased parasympathetic reactivity predicts focused attention^12–14^. Yet it remains unclear whether partners talking were mutually relaxed or their attention was mutually divided over social engagement and the task they worked on. Likewise, there is no certainty that partners cooperating were mutually focused on the same singular aspect of their experience together. Our inability to draw inferences from physiology to mental state is a limitation of the current research as it is for much of the prior work on physiological synchrony. Yet these findings point to critical extensions for future research. Given that so much theorizing has tended toward conceptualizing physiological synchrony as proximal to mental synchrony, it is imperative that future research is designed to fully test this assumption by including measures known to reflect mental states associated with shifts in autonomic activity.

It is unclear why sympathetic and parasympathetic synchrony differentially predicted measures of affiliation beyond the fact that shifts in sympathetic and parasympathetic reactivity correspond to different mental states. Importantly, the measures of affiliation included in this study do not exhaust all possible social outcomes that might reasonably correspond to physiological synchrony in the autonomic nervous system, nor did we even consider other psychological outcomes like performance or cognition. Moreover, as highlighted above, tackling the extent to which physiological synchrony corresponds to the sharing of specific mental states might help scholars to better determine when synchrony should predict specific outcomes. Despite these limitations, our work demonstrates the value of studying physiological synchrony between partners in each branch of the autonomic nervous system as well as the underlying patterns of arousal partners share.

We also observed that some stranger pairs displayed discordant patterns of sympathetic synchrony—as one partner’s sympathetic arousal increased their partner’s arousal decreased. Prior work posits that turn-taking might influence whether partners display discordant synchrony^16,18^. During conversations, for example, partners might display discordant parasympathetic synchrony as they take turns talking because talking can influence parasympathetic nervous system activity via respiration. In the current study, however, talking moderated neither sympathetic nor parasympathetic synchrony. Thus, other unexplored factors shaped whether partners displayed concordant versus discordant sympathetic synchrony.

An important limitation of the present research is that the no talking condition predicted significantly more sympathetic activity during baseline than the talking condition (see Supplementary Information). Thus, baseline sympathetic activity is *not* independent of experimental condition. To address this issue, we modeled residualized change, which examines change in sympathetic and parasympathetic activity beyond baseline levels, while taking measurement error into account. Using residualized change scores can help lessen the impact of the dependency between condition and baseline sympathetic activity on our overall results. Nonetheless, our results should be interpreted within the context of this limitation.

We gain clarity into the meaning of physiological synchrony by considering it in concert with other pieces of knowledge about the situation. Indeed, our findings underscore the importance of thinking beyond physiological synchrony *per se* to consider contextual factors in descending order of proximity: What social context do we find ourselves in? How well do we know our partner? What physiological systems are being affected and, as the current work emphasizes, how reactive are these systems? In the absence of acquaintanceship processes (e.g., talking or cooperating with a partner) and physiological reactivity, there may not be as much psychosocial relevance to physiological synchrony. While there is still much to understand about physiological synchrony, the present study demonstrates that physiological synchrony in the context of social interaction between strangers partially reflects social processes.

Taken together, our work shows that features of a social interaction cause levels of physiological synchrony that go beyond aspects of a shared environment or common task. Conversation and cooperation elicited the greatest sympathetic reactivity whereas conversation and competition elicited the greatest parasympathetic reactivity. Thus, even though the magnitude of physiological synchrony displayed did not vary by features of the social context, the patterns of arousal underlying physiological synchrony did. We also found evidence that social context and physiological reactivity can contextualize the meaning of physiological synchrony in the parasympathetic nervous system in ways that are fundamental to understanding the relationship between physiological synchrony and psychosocial constructs. Although this finding was not confirmed for sympathetic synchrony, the current work suggests that simple observation of physiological synchrony can be insufficient for inferring social processes.

## Methods

### Participants and procedures

#### Design

The experiment was a 2 (talking or no talking) × 2 (cooperative or competitive) dyadic design. Analyses of physiological synchrony included ten observations for each participant (our measure of physiological synchrony was created by covarying ten 30-second segments of physiological data from the 5-minute interaction).

#### Assessment of sample size and recruitment

Prior to data collection, we used the *ANOVA: Repeated Measures, within-between interaction* statistical test in G*Power (version 3.1.3) to derive our sample size^19^. We set the probability of Type I Error set to 0.05, statistical power to 0.80, and had an anticipated effect size of *r* = 0.14^10^. Correlation among repeated measures and nonsphericity corrections were set to G*Power’s default values. This test determined the need to collect data from at least 64 participants in total and the actual power reported by this test was 0.81.

#### Procedures

Introductory Psychology students and community members from the University of Toronto (*N* = 136) participated in a 2-hour study in same-sex, same-ethnicity pairs. We accidentally scheduled one cross-gender pair. This pair was removed from analyses leaving a total sample of 134 participants. The sample was 70% female and the mean age was 20.53 years (*SD*_*age*_ = 5.66). The ethnic composition of this sample was diverse: 2% Black, 36% East Asian, 1% Hispanic, 4% Middle Eastern, 2% Multi-ethnic, 14% South Asian, 8% South East Asian, 2% West Indian, and 31% White. Participants received two course credits, $20.00, or a combination of credits and money, in addition to a performance bonus. The study was approved by the University of Toronto Research Ethics Boards (REB), all methods were carried out in accordance with the guidelines and regulations set out by the REB, and all participants provided informed consent.

Participants learned that the purpose of the study was to “examine how people embody perceptions of their social environments.” Participants completed an online survey prior to the experimental lab session. We did not analyze any pretest variables. Moreover, a number of subjective measures collected in the lab were not analyzed for the current paper. A full list of subjective measures collected is provided in the Protocol Exchange. After giving consent, participants orally answered questions about their health and health-related behaviours. Then, participants were connected to physiological recording devices. The experimenters calibrated the physiological signals before participants sat for a five-minute baseline recording.

After the baseline recording, participants completed an online survey that included the pre-interaction measures of negative and positive affect. Then, participants were introduced to their partner and the experimental task was explained. Pairs were randomly assigned to complete one of four tasks that varied along two social-contextual factors: 2 (talking versus no talking) × 2 (cooperative versus competitive). Task details are described in greater detail in the next section. After completing the social-contextual task, participants were separated in order to record their physiological recovery from the task. Participants then completed another set of surveys prior to a full debriefing.

#### Experimental primes

Across conditions, participants completed identical tasks. Participants each received a three-foot string in which to tie knots using only one hand (i.e., the dominant hand) over a five-minute period. The only difference across conditions was how the task was framed.

Talking-cooperative. Pairs in the talking-cooperative condition were given the following instructions:

We want you to get to know your partner while working toward a collaborative goal. You will both receive one long string in your dominant hand. We want you to tie as many knots as possible in five minutes on one long string using one hand. This is a fun party-game, designed to encourage social affiliation and cooperation. You should be trying to get to know each other at the same time as completing this task so feel free to ask each other personal questions and at the same time try to cooperate with each other on this task. The more knots you can tie as a team, the more points you will each receive. The team with the highest points will be entered into a draw to receive two $50.00 gift cards at Amazon.ca.

Talking-competitive. Pairs in the talking-competitive condition were given the following instructions:

We want you to get to know your partner while competing for points/rewards. You will both receive one long string in your dominant hand. We want you to tie as many knots as possible in five minutes on one long string using one hand. This is a fun game, designed to encourage social affiliation and competition. You are competing against each other for a small reward, but you should try to get to know each other at the same time so feel free to socialize and to ask each other personal questions. The more knots you can tie as an individual, the more points you will receive. Whoever has the most points will be entered into a draw to receive a $50.00 gift card at Amazon.ca.

No talking-cooperative. Pairs in the no talking-cooperative condition were given similar instructions as participants in the talking-cooperative condition, with the exception that they were instructed not to talk:

We want you to cooperate on a collaborative goal without having a conversation. You will both receive one long string in your dominant hand. We want you to tie as many knots as possible in five minutes on one long string using one hand. Do not socialize, do not talk. Just work on the task. The more knots you can tie as a team, the more points you will each receive. The teams with the highest points will be entered into a draw to receive two $50.00 gift cards at Amazon.ca.

No talking-competitive. Likewise, pairs in the no talking-competitive condition were given similar instructions as participants in the no talking-cooperative condition, with the exception that they were instructed not to talk to each other:

We want you to compete for points/rewards on task without having a conversation. You will both receive one long string in your dominant hand. We want you to tie as many knots as possible in five minutes on one long string using one hand. Do not socialize, do not talk. Just work on the task. You are competing against each other. Whoever has the most points at the end will be entered into a draw to receive a $50.00 gift card at Amazon.ca.

### Physiological data

#### Sympathetic nervous system activity

Sympathetic nervous system activity was measured with Cardiac Sympathetic Index (CSI)^20^. CSI is a validated measure that uniquely reflects sympathetic activity, not parasympathetic activity. That is, CSI is unaffected by pharmacological interventions intended to block parasympathetic activity but decreases in healthy adults when a sympathetic blockade is administered^20^.

#### Parasympathetic nervous system activity

Parasympathetic nervous system activity was measured with Respiratory Sinus Arrhythmia (RSA). RSA represents oscillations in heart rate variability due to the influence of patterns of respiration. These patterns of respiration reflect a parasympathetic influence on the heart through the vagus nerve^12^. RSA can be measured noninvasively and is considered predominantly a measure of parasympathetic activation^21^. RSA values tend to be non-normally distributed. Thus, the log value is typically derived (logRSA). Increases in logRSA indicate increased vagal influence and, thus, increased parasympathetic activation.

### Data acquisition and processing

Cardiovascular data, from which sympathetic and parasympathetic activity were estimated, were measured using electrocardiograph (ECG), which records ventricular contraction. ECG was recorded with electrodes arranged in the modified Lead II placement and sent to a computer through a Biopac ECG100C Module and MP150 amplifier (Biopac Systems, Inc., Goleta, California). Data was continuously acquired and monitored using AcqKnowledge version 4.4 (BiopacSystems, Inc., Goleta, California) at a sampling rate of 1000 Hz.

ECG recordings were also scored using Acknowledge version 4.4 (Biopac Systems, Inc., Goleta, California). Nineteen research assistants, trained by the lead author, visually inspected ECG waveforms to identify and score the R wave, which represents early depolarization of the ventricles. Research assistants were instructed to set aside files with problematic waveforms, which the lead author scored. Interbeat intervals (IBI), the time to complete one heart cycle (in milliseconds) were exported from the scored data into spreadsheets for further examination. Specifically, an in-house computer algorithm examined the spreadsheets for improbable IBIs. The algorithm read each row of IBI data, setting the current IBI as a target IBI, which was then compared to the subsequent IBI value. If the target IBI was 0.6 the size of the subsequent IBI, the algorithm flagged the target IBI as questionable and entered its location in time and the name of the target file into a spreadsheet. This spreadsheet was then used by the lead author to pinpoint the precise location of questionably placed R waves for further visual inspection and, if necessary, correction.

#### Derivation of sympathetic and parasympathetic activity

The IBI data were prepared for upload to CMetX Software, a freely available suite of digital tools for estimating CSI and logRSA values^22^. To be consistent with the physiological synchrony literature^23^, we planned to examine synchrony in phasic changes in sympathetic and parasympathetic activity across concurrent, 30-second segments of CSI and logRSA values. An in-house computer algorithm segmented the IBI data into 54-second epochs because CMetX truncates the first and the final twelve seconds of the input. CMetX estimated logRSA by deriving respiration from the IBI using a 0.12–0.40 Hz bandpass filter at a sampling rate of 10 Hz. This filter corresponds to a common respiration range in adult humans^24^.

### Subjective measures of affiliation

#### Perceived similarity

Participants rated how similar they perceived their partner to be using five face-valid items including, “My partner and I are very similar”, “There are many parallels between me and my partner”, “My partner and I share a lot in common”, “My partner and I think alike”, and “Although we did not get to know each other very well, I get the sense that my partner and I share similar attitudes.” Items were rated on a scale from 1 (*strongly disagree*) to 7 (*strongly agree*) and the scale internal consistency was excellent (*α* = 0.92, *M* = 4.41, *SD* = 0.97).

#### Friendship interest

Participants indicated their friendship interest by responding to the following four face-valid items, “How much do you like your partner?”, “How likely is it that you would become friends with your partner?”, “How much would you want to interact with your partner in the future?”, and “How much did you enjoy interacting with your partner?” Items were rated on a scale from 1 (*not at all*) to 7 (*very*). The scale had excellent internal reliability (*α* = 0.92*, M* = 4.81*, SD* = 1.08).

#### Changes in positive and negative affect

Participants indicated their current levels of positive and negative affect immediately before and after their interaction using the Positive and Negative Affect Schedule (PANAS)^25^. Ten items measured positive affect, including “Right now how interested do you feel?”, “…how excited do you feel?”, and “…how strong do you feel?” Ten items measure negative affect, including “Right now how distressed do you feel?”, “…how upset do you feel?”, and “…how guilty do you feel?” Items were rated on a scale from 1 (*not at all*) to 5 (*extremely*). The scale had excellent internal reliability (pre-positive affect: *α* = 0.92*, M* = 2.73*, SD* = 0.83; post-positive affect: *α* = 0.91*, M* = 2.57*, SD* = 0.84; pre-negative affect: *α* = 0.86*, M* = 1.53*, SD* = 0.54; post-negative affect: *α* = 0.90*, M* = 1.28*, SD* = 0.46).

### Behavioural measure of affiliation

#### Friendship initiation

Each participant and their partner had an opportunity to exchange their email addresses. Participants and their partner were told, “If you enjoyed your interaction and would be willing to see this person in the future, then please enter your email address below. We will only exchange your contact information if both you and your partner agree to exchange this information.” A majority of participants shared their email address with their partner (58.96%).

### Analytic approach

All analyses used multilevel modeling. Multilevel models were run using the nlme package^26^ for the statistical package R (version 3.4.2)^27^. Models were estimated with an unstructured covariance matrix and degrees of freedom were estimated using the between-within method. Partial effect sizes are provided as correlation coefficients, converted from the *t*-statistic and degrees of freedom associated with each slope^28^. Simple effects were probed within each relevant condition and at +/− 1 *SD* for sympathetic reactivity using the emmeans package in R to apply the Tukey adjustment to the *p*-values for each comparison^29^. Thus, only the adjusted *p*-values are reported for simple effects. Full details of our analytic approach, including handling of missing data can be found in Supplementary Information.

## Supplementary information

Supplementary Information

## Data Availability

The data that support the findings of this study are available on request from the corresponding author (C. D.). The data are not publicly available because consent to make these data public was not obtained from participants a priori and thus we do not have ethics approval to post it online.

## Code Availability

All syntax codes used for data organization, analyses, and modeling are available: https://osf.io/yjmrw/?view_only=9e9223c693004b96ae217e4c0ab28c67.

## References

[1] Moiseff A, Copeland J (2010). Firefly synchrony: A behavioral strategy to minimize visual clutter. Science..

[2] Perrot C (2016). Sexual display complexity varies non-linearly with age and predicts breeding status in greater flamingos. Sci. Rep..

[3] Bulla M (2016). Unexpected diversity in socially synchronized rhythms of shorebirds. Nature.

[4] McClintock MK (1971). Menstrual synchrony and suppression. Nature.

[5] Hodge SJ, Bell MBV, Cant MA (2011). Reproductive competition and the evolution of extreme birth synchrony in a cooperative mammal. Biol. Lett..

[6] Levenson RW, Gottman JM (1983). Marital interaction: Physiological linkage and affective exchange. J. Pers. Soc. Psychol..

[7] Ekman I (2011). Social interaction in games: Measuring physiological linkage and social presence. Simul. Gaming.

[8] Chanel G, Kivikangas JM, Ravaja N (2012). Physiological compliance for social gaming analysis: Cooperative versus competitive play. Interact. Comput..

[9] Palumbo RV (2017). Interpersonal autonomic physiology: A systematic review of the literature. Personal. Soc. Psychol. Rev..

[10] Danyluck, C. & Page-Gould, E. Intergroup dissimilarity predicts physiological synchrony and affiliation in intergroup interaction. *J. Exp. Soc. Psychol*. **74** (2018).

[11] Kelsey Robert M. (2012). Beta-adrenergic cardiovascular reactivity and adaptation to stress: The cardiac pre-ejection period as an index of effort. How motivation affects cardiovascular response: Mechanisms and applications.

[12] Porges SW (2001). The polyvagal theory: Phylogenetic substrates of a social nervous system. Int. J. Psychophysiol..

[13] Cacioppo, J. T., Sandman, C. A. & Walker, B. B. The effects of operant heart rate conditioning on cognitive elaboration and attitude change. *Psychophysiology* 330–338 (1978). 10.1111/j.1469-8986.1978.tb01389.x 693741

[14] Suess PE, Porges SW, Plude DJ (1994). Cardiac vagal tone and sustained attention in school‐age children. Psychophysiology.

[15] Kaplan HB, Burch NR, Bloom SW, Edelberg R (1963). Affective Orientation and Physiological Activity (GSR) in Small Peer Groups. Psychosom. Med..

[16] Reed RG, Randall AK, Post JH, Butler EA (2013). Partner influence and in-phase versus anti-phase physiological linkage in romantic couples. Int. J. Psychophysiol..

[17] Brambilla M, Sacchi S, Menegatti M, Moscatelli S (2016). Honesty and dishonesty don’t move together: Trait content information influences behavioral synchrony. J. Nonverbal Behav..

[18] Vallacher RR, Nowak A, Zochowski M (2005). Dynamics of social coordination: The synchronization of internal states in close relationships. Interact. Stud..

[19] Franz, F., Erdfelder, E., Lang, A.-G. & Buchner, A. G*Power 3: A flexible statistical power analysis program for the social, behavioral, and biomedical sciences. *Behav. Res. Methods*. 175–191 (2007). 10.3758/bf03193146 17695343

[20] Toichi M, Sugiura T, Murai T, Sengoku A (1997). A new method of assessing cardiac autonomic function and its comparison with spectral analysis and coefficient of variation of R-R interval. J. Auton. Nerv. Syst..

[21] Blascovich, J., Mendes, W. B., Vanman, E. & Dickerson, S. S. *Social Psychophysiology for Social and Personality Psychology*. (SAGE Publications, 2011).

[22] Allen JJB, Chambers AS, Towers DN (2007). The many metrics of cardiac chronotropy: A pragmatic primer and a brief comparison of metrics. Biol. Psychol..

[23] Helm, J. L., Sbarra, D. A. & Ferrer, E. Coregulation of respiratory sinus arrhythmia in adult romantic partners. *Emotion* **14** (2014). 10.1037/a0035960 24708502

[24] Camm AJMM (1996). Heart rate variability: standards of measurement, physiological interpretation and clinical use. Task Force of the European Society of Cardiology and the North American Society of Pacing and Electrophysiology. Circulation.

[25] Watson D, Clark LA, Tellegen A (1988). Development and validation of brief measures of positive and negative affect: The PANAS Scales. J. Pers. Soc. Psychol..

[26] Pinheiro, J., Bates, D., DebRoy, S., Sarkar, D. & R Core Team. nlme: Linear and Nonlinear Mixed Effects Models. *R package version 3.1*, https://CRAN.R-project.org/package=nlme (2017).

[27] R Core Team. R: A language and environment for statistical computing. *R Foundation for Statistical Computing, Vienna, Austria*, https://www.R-project.org/ (2017).

[28] Kashdan TB, Steger MF (2006). Expanding the topography of social anxiety. Psychol. Sci..

[29] Lenth R (2019). emmeans: Estimated Marginal Means, aka Least-Squares Means. R package version 1.2.4..

