# Author Correction: Social and Physiological Context can Affect the Meaning of Physiological Synchrony

**DOI:** 10.1038/s41598-019-48223-z

**Published:** 2019-08-15

**Authors:** Chad Danyluck, Elizabeth Page-Gould

**Affiliations:** 1 Centers for American indian and Alaska Native Health, Colorado School of Public Health, University of Colorado Denver Anschutz Medical Campus, Denver, USA; 2 Department of Psychology, University of Toronto, Toronto, Canada

Correction to: *Scientific Reports* 10.1038/s41598-019-44667-5, published online 03 June 2019

In Figures 1b and 2b, the x-axes are incorrectly labelled. The correct Figures 1 and 2 appear below.

**Figure 1:**
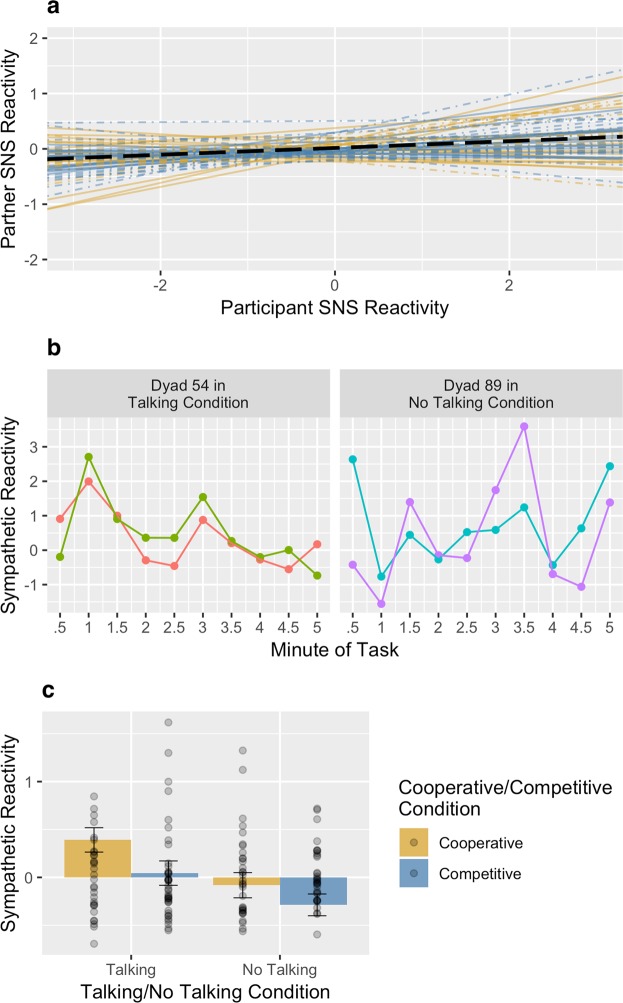
Reliability of the per-pair slope estimates for sympathetic synchrony (**a**). Sympathetic synchrony during the knot-tying task for two example pairs (**b**). Effect of experimental conditions on sympathetic reactivity (**c**). Error bars represent standard errors of the estimated marginal means.

**Figure 2:**
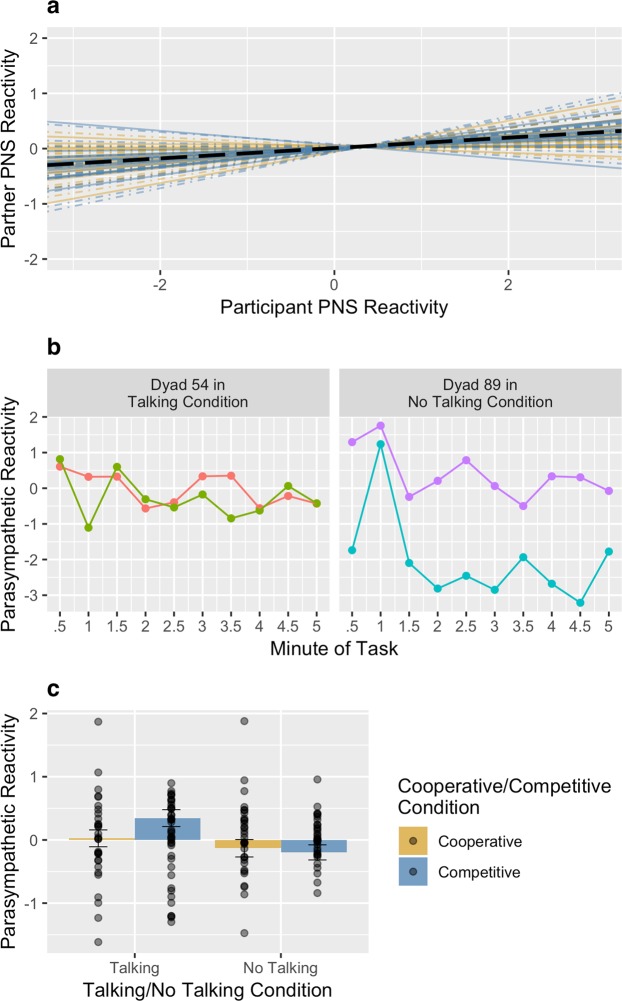
Reliability of the per-pair slope estimates for parasympathetic synchrony (**a**). Parasympathetic synchrony during the knot-tying task for two example pairs (**b**). Effect of experimental conditions on parasympathetic reactivity (**c**). Error bars represent standard errors of the estimated marginal means.

