# Supplementary material for: Social and Physiological Context can Affect the Meaning of Physiological Synchrony
Source: Sci Rep. 2019 Jun 3;9:8222. doi: 10.1038/s41598-019-44667-5 (PMC6547677; doi:10.1038/s41598-019-44667-5)
Supplement: Supplementary file 1 — Supplementary Information [file 41598_2019_44667_MOESM1_ESM.docx]

**Social and Physiological Context can Affect the Meaning of Physiological Synchrony**

**Supplementary Information**

Chad Danyluck^1^ and Elizabeth Page-Gould^2^

^1^Centers for American Indian and Alaska Native Health, Colorado School of Public Health, University of Colorado Denver

^2^Department of Psychology, University of Toronto

Correspondence and requests for materials should be addressed to C. D. (email: chad.danyluck@ucdenver.edu)

# Supplementary Information

## Missing Data Handling

Although we had a mostly complete dataset, approximately 0.81% of our data were missing. Moreover, a Little Test revealed that the missing data patterns were not missing completely at random. Thus, an ideal way to handle the missing data was to use imputation (Rubin, 1996). However, we encountered two challenges: Although we had a three-level dataset for the initial physio analyses – compared to a 2-level dataset for our exploratory analyses of social outcomes – we did not have enough data at the first level to conduct a 3-level imputation (i.e., considering participants within dyads), because we only had measured sympathetic and parasympathetic activity at the first level and their patterns of missingness were identical, given that they were derived from the same measurement device. Therefore, we imputed the missing values in our dataset as a 2-level dataset using the “2l.norm” imputation method in the mice multiple imputation package (van Buuren & Groothuis-Oudshoorn, 2011), where we accounted for nesting within dyads. This created a second challenge insofar as we could not then iteratively run our primary analyses on multiple imputed datasets. So, we used the complete() function of the mice package to create one complete dataset from the values imputed during one of the imputations. Ultimately, this means that we imputed missing values from linear models derived from the non-missing values, using a method that allowed for heteroskedasticity in the data.

**Main Analyses**

Sympathetic synchrony and parasympathetic synchrony can be observed through the covariation of CSI and logRSA values, respectively, measured from two people over time (our exact model is depicted in the multilevel equations reported in the main text and the derivation of CSI and logRSA is described in the extended methods). To account for individual differences in sympathetic and parasympathetic activity, however, and to be consistent with the physiological synchrony literature, changes from baseline in sympathetic and parasympathetic activity were first calculated (i.e., reactivity scores were created) prior to analysis.^1,2^ Specifically, CSI and logRSA values estimated during the social interaction were regressed on the last half of the baseline CSI and logRSA values, respectively, to create “residualized” change scores (i.e., change scores that assume some measurement error). Although it is often common to create residualized change scores against the final minute of baseline^1^, some of our participants had missing data in the final segments of the baseline recording and, thus, we included a larger segment of the baseline period in our change scores (i.e., the last half of baseline) so as to include more data observations. T-tests confirmed that differences between mean baseline values in the last half of the baseline recording period and the last minute were not significantly different: CSI, *M_lasthalf_* = 2.39, *M_lastminute_* = 2.35, *t*(245.95) = 0.32, *p* = 0.746; logRSA, *M_lasthalf_* = 6.04, *M_lastminute_* = 6.11, *t*(259.32) = −0.49, *p* = 0.625.

## Identifying the Best Random Effects Structure

A reviewer wisely pointed out that we needed to account for moving averages in our models and suggested that we include the partner’s values from the previous time point as an additional predictor in our model. This is why the models for both sympathetic and parasympathetic synchrony included the fixed effects of the partner’s lagged physiological responses (“lagged partner”) and the participant’s concurrent physiological responses (“synchrony”), with our two experimental manipulations of the degree of social interaction and the competitive or cooperative context of the knot-tying task as dyad-level moderators of synchrony. The purpose of including the partner’s lagged physiological responses as a predictor was so that synchrony would be estimated above and beyond what would be expected from the partner’s physiology at the previous time point. Given that both the lagged partner and synchrony terms were measured within-subjects (i.e., at each minute of the task), the typical approach to the random effects would be to include the maximal random effects structure for these within-subjects variables.^3^ Specifically, the maximal random effects structure for our data would be:

$partner_{ijk}=\pi_{0_{jk}}+\pi_{1_{jk}}laggedpartner_{ijk}+\pi_{2_{jk}}synchrony_{ijk}+e_{ijk}$ (1)

$\pi_{0_{jk}}=\beta_{{00}_{k}}+r_{0_{jk}}$ (2)

$\pi_{1_{jk}}=\beta_{{10}_{k}}+r_{1_{jk}}$ (3)

$\pi_{2_{jk}}=\beta_{{20}_{k}}+r_{2_{jk}}$ (4)

$\beta_{{00}_{k}}=\gamma_{000}+\gamma_{001}talking_{k}+\gamma_{002}cooperation_{k}+\gamma_{003}talking_{k}cooperation_{k}+u_{0_{k}}$ (5)

$\beta_{{10}_{k}}=\gamma_{100}+u_{1_{k}}$ (6)

$\beta_{{20}_{k}}=\gamma_{200}+\gamma_{201}talking_{k}+\gamma_{202}cooperation_{k}+\gamma_{203}talking_{k}cooperation_{k}+u_{2_{k}}$ (7)

In the above, equations, the subscripts refer to each level of the data, where *i* is each minute that physio was measured during the knot-tying task, *j* is each individual participant, and *k* indicates each dyad. In all equations, the variables, $partner_{ijk}$ refers to the partner’s physiological reactivity, $laggedpartner_{ijk}$ refers to the partner’s physiological reactivity at the previous minute, $synchrony_{ijk}$ refers to the participant’s physiological reactivity measured at the same time as the physio in the $partner_{ijk}$ variable, $talking_{k}$ refers to whether the dyad was assigned to interact (coded with 1) or not interact (coded with −1), $cooperation_{k}$ refers to whether the dyad was given instructions that made the knot-tying task cooperative (coded with 1) or competitive (coded with −1).

All parameters represented with a $\pi$ are intercepts and slopes that were estimated uniquely for each participant and all variables represented with a $\beta$ are intercepts and slopes estimated uniquely for each dyad. If a parameter has the subscript of 0, then it is an intercept, and all other subscripts (i.e., 1 through 3) represent slopes in the order that they appear in each equation. In Equation 1, $e_{ijk}$ is the residual term at the lowest level (i.e., the deviations that cannot be explained by the rest of the model). Each parameter represented by a $\pi$ or $\beta$ are themselves estimated, which is why they each have their own equations with their own intercepts, slopes, and residuals. Specifically, since each of those parameters are estimated, we assume that each one is estimated with some error, so each of their equations have residual terms to represent that error, which are denoted with an $r$ for the participant-level equations (i.e., Equations 2 – 4 that estimated a parameter denoted with a $\pi$) and with an $u$ for dyad-level equations (i.e., Equations 5 – 7 that estimated a parameter denoted with a $\beta$). Finally, the $\gamma$ symbols in the dyad-level equations (Equations 5 – 7) represent the intercept for each parameter (i.e., its average value across dyads) and the slopes of dyad-level predictors (i.e., the experimental conditions). There are no slopes in the participant-level equations (i.e., the ones predicting $\pi$ parameters), because we had no participant-level predictors.

These multilevel equations can be collapsed into a single equation in mixed model format:

$$\begin{matrix} partner_{ijk}=\gamma_{000}+\gamma_{100}laggedpartner_{ijk}+\gamma_{200}synchrony_{ijk}+\gamma_{001}talking_{k}+\gamma_{002}cooperation_{k} \\ +\gamma_{003}talking_{k}cooperation_{k}+\gamma_{201}talking_{k}synchrony_{ijk}+\gamma_{202}cooperation_{k}synchrony_{ijk} \\ +\gamma_{203}talking_{k}cooperation_{k}synchrony_{ijk} \\ +u_{0_{k}}+u_{1_{k}}laggedpartner_{ijk}+u_{2_{k}}synchrony_{ijk} \\ +r_{0_{jk}}+r_{1_{jk}}laggedpartner_{ijk}+r_{2_{jk}}synchrony_{ijk} \\ +e_{ijk} (8) \end{matrix}$$

However, specifying the maximal random effects resulted in a non-positive definite Hessian matrix for both our sympathetic and parasympathetic models. A non-positive definite Hessian matrix implies that the model is overspecified or otherwise too complex for these data. It also implies that there may be no variance in one of the random effects. This is not surprising, because the maximal model allowed for different slopes of $synchrony_{ijk}$ and $laggedpartner_{ijk}$ at the individual level, but these slopes should be invariant within dyads. Furthermore, the reason the reviewer suggested that we include the lagged partner effect was to account for serial dependency—albeit moving averages—but we would also expect to see this in an autocorrelated covariance structure. Therefore, we decided to identify the ideal random effects specification by comparing 8 models that varied in 3 ways in the specification of random effects: (a) including random slopes for both $synchrony_{ijk}$ and $laggedpartner_{ijk}$ or only $synchrony_{ijk}$; (b) including random slopes at both the individual and dyad levels or only the dyad level; (c) using an unstructured or autoregressive correlation matrix. All models had random intercepts specified for both the individual and dyad levels.

To compare the models, we used the AIC values for each model to calculate Akaike weights, which were then used to create a candidate set of the most probable models, given the data and the other models tested.^4^ Note that more than one model can end up in the candidate set, in which case we decided to select the simplest model (i.e., that had the fewest degrees of freedom) as our final model from the candidate set.

**Sympathetic Synchrony Model.** Supplementary Table S3 shows the results of the model comparison for the models predicting partner sympathetic reactivity. After calculating Akaike weights, two models remained candidates for the final analysis. Both candidate models used an autoregressive covariance structure and only included random slopes at the dyad level, but they differed in whether a random slope for $laggedpartner_{ijk}$ was estimated. We selected the model that only included a random slope for $synchrony_{ijk}$, *df* = 15, over the model with random slopes for both $synchrony_{ijk}$ and $laggedpartner_{ijk}$, *df* = 18, because it was the simplest model in the candidate set.

**Parasympathetic Synchrony Model.** Supplementary Table S4 shows the results of the model comparison for the models predicting partner parasympathetic reactivity. One of the models failed to converge, specifically the model that specified random slopes for both $synchrony_{ijk}$ and $laggedpartner_{ijk}$ at both the individual and dyad levels using an autoregressive covariance matrix. So, it was not included in the model comparison. Nonetheless, the comparison of parasympathetic synchrony models suggested that the same random effects structure that was ideal for the sympathetic synchrony model was also ideal for the parasympathetic synchrony model. After calculating Akaike weights, two models remained candidates for the final analysis. Both candidate models used an autoregressive covariance structure and only included a random slope for $synchrony_{ijk}$, but they differed in whether the random slope for $synchrony_{ijk}$ was estimated at both the individual and dyad levels, *df* = 17, or only at the dyad level, *df* = 15. The simplest model was the one that only included a random slope for $synchrony_{ijk}$ at the dyad level, so that was the model we used in our final analyses.

**Baseline Autonomic Nervous System Activity**

We checked for possible differences in baseline sympathetic and parasympathetic activity across conditions. To do this, baseline CSI and logRSA values were individually regressed on the interaction between talking/no talking and cooperative/competitive conditions with a random intercept for dyad. These tests revealed that participants assigned to the talking condition had higher baseline sympathetic activity, *b* = −0.14, *SE* = 0.07, 95% CI [−0.288, −0.005], *t*(63) = −2.07, *p* = .042, *r* = −0.253 than the other conditions. Thus, baseline sympathetic activity is *not* independent of experimental condition. There were no other significant effects for sympathetic activity at baseline (Table S5) and baseline parasympathetic activity was not predicted by experimental conditions, *b* = 0.13, *SE* = 0.10, 95% CI [−0.083, 0.335], *t*(63) = 1.21, *p* = .232, *r* = 0.150 (Table S5).

It is standard procedure in psychophysiology to control for baseline values when predicting values measured at subsequent time points, such that physiological responses are considered relative to baseline. To address the fact that baseline sympathetic activity was dependent on condition, we modeled residualized change, which examines change in sympathetic and parasympathetic activity beyond baseline levels, while taking measurement error into account. Using residualized change scores can help lessen the impact of the dependency between condition and baseline sympathetic activity on our overall results.

## Reactivity and Social Processes

## Perceived similarity.

***Sympathetic Nervous System.*** Similar to the results for sympathetic synchrony, sympathetic reactivity also predicted greater perceived similarity with one’s partner, *b* = 0.479, *SE* = 0.177, 95% CI [0.153, 0.805], *t*(288) = 2.716, *p* = 0.007, *r* = 0.158 (Figure S1).

The main effect for sympathetic reactivity was qualified by two higher-order 2-way interactions with the talking conditions (talking/no talking × sympathetic reactivity, *b* = 0.457, *SE* = 0.177, 95% CI [0.131, 0.783], *t*(288) = 2.591, *p* = 0.01, *r* = 0.151) and the cooperative/competitive conditions (cooperation/competition × sympathetic reactivity, *b* = −0.364, *SE* = 0.177, 95% CI [−0.69, −0.038], *t*(288) = −2.062, *p* = 0.04, *r* = −0.121), but these effects were not further crossed in a higher order 3-way interaction, *b* = −0.289, *SE* = 0.177, 95% CI [−0.615, 0.037], *t*(288) = −1.639, *p* = 0.102, *r* = −0.096.

When participants were talking with each other, sympathetic reactivity predicted greater perceived similarity, *adj. p* = 0.003 (Figure S2, panel a). When breaking down the 2-way interaction with cooperation/competition and sympathetic reactivity (Figure S2, panel b), the reliable difference was that sympathetic reactivity predicted more perceived similarity among participants who were competing with their partners, *adj. p* = 0.008.


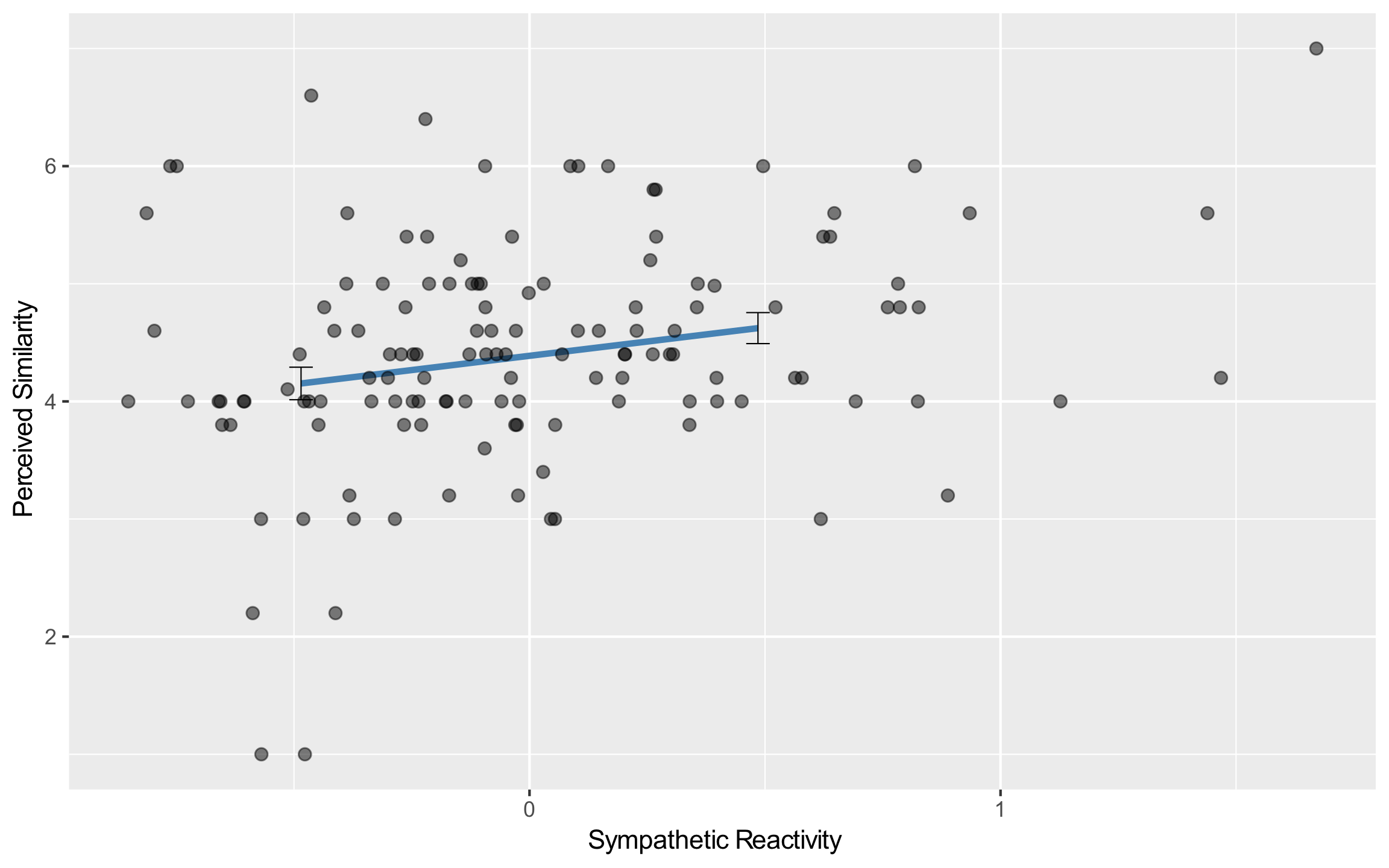


**Figure S1.** Association between sympathetic reactivity and perceived similarity. Error bars represent standard errors of the estimated marginal means.


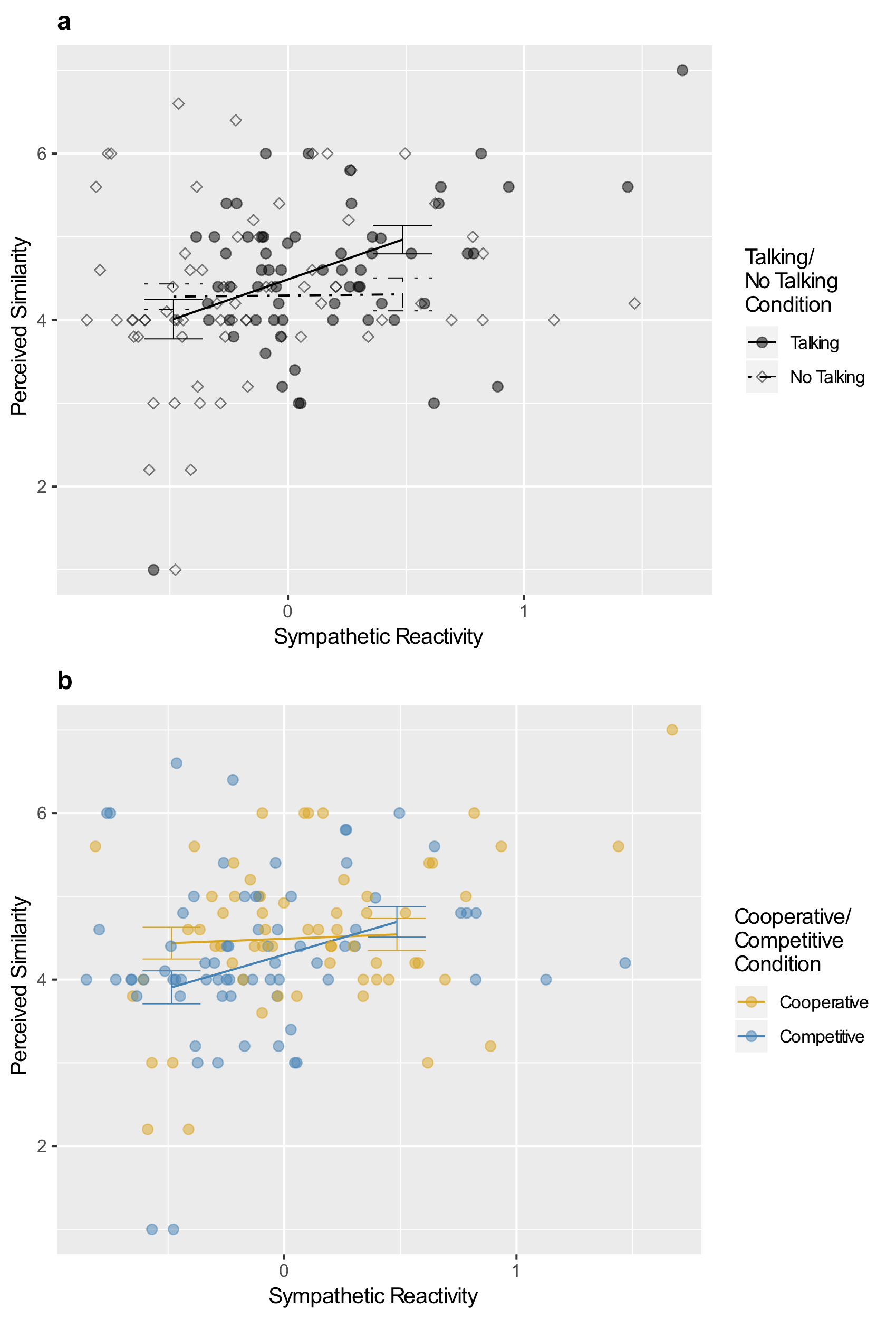


### **Figure S2.** Association between sympathetic reactivity and perceived similarity according to conditions (talking condition, panel a; cooperative condition, panel b). Error bars represent standard errors of the estimated marginal means.

***Parasympathetic Nervous System.*** In the parasympathetic nervous system, perceived similarity was predicted by a 3-way interaction between the talking conditions, cooperative/competitive conditions, and parasympathetic reactivity, *b* = −0.429, *SE* = 0.129, 95% CI [−0.668, −0.19], *t*(288) = −3.317, *p* = 0.001, *r* = −0.192. After adjusting for multiple comparisons, the only reliable effect was that parasympathetic reactivity predicted less perceived similarity among participants who were competing with each other while not directly interacting, *adj. p* = 0.021 (Figure S3).


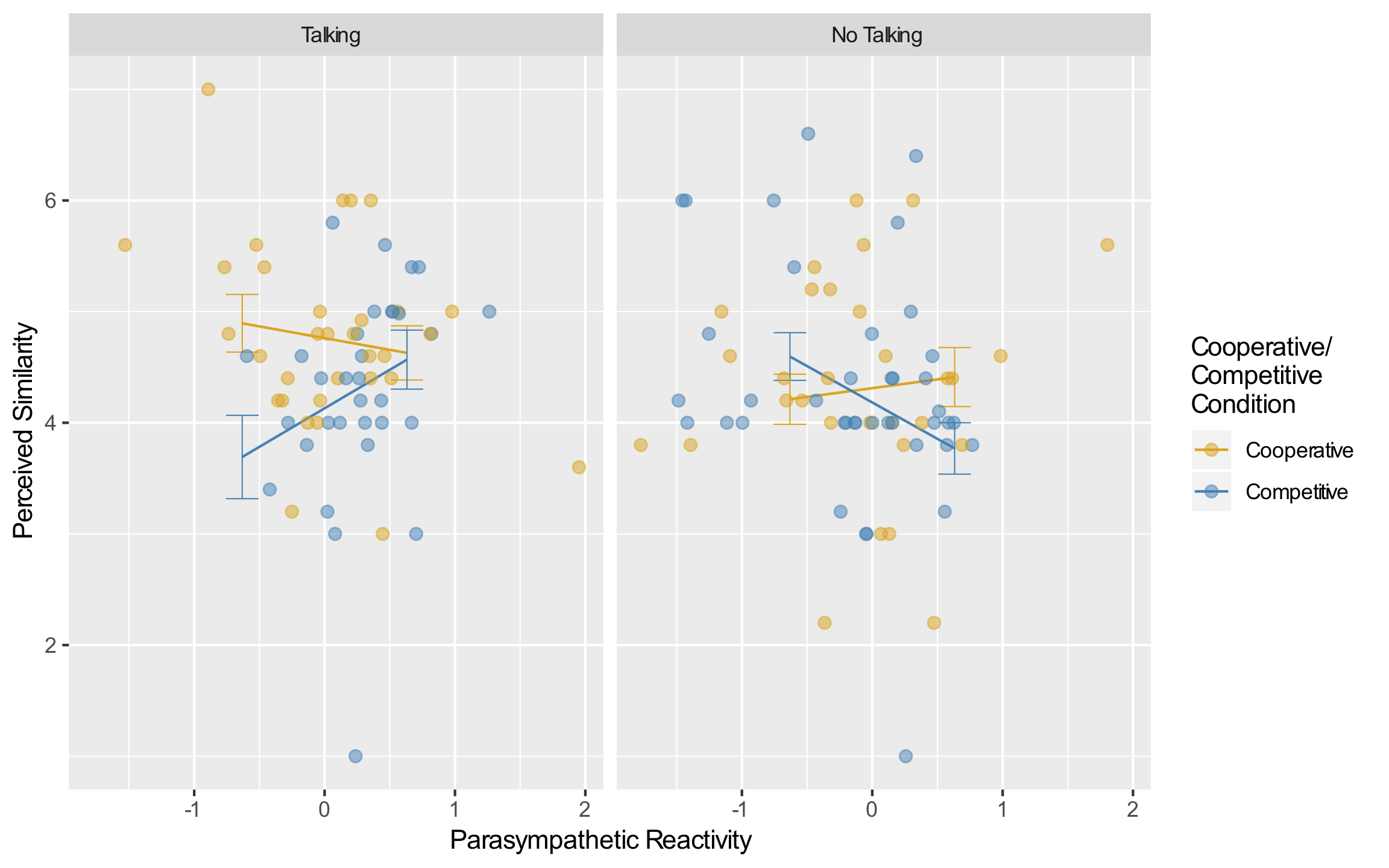


### **Figure S3.** 3-way interaction between talking conditions, cooperative/competitive conditions, and parasympathetic reactivity predicting perceived similarity.

##

## Friendship Interest.

***Sympathetic Nervous System.*** There was a main effect for sympathetic reactivity to predict greater friendship interest, *b* = 0.694, *SE* = 0.19, 95% CI [0.343, 1.046], *t*(288) = 3.65, *p* < .001, *r* = 0.21, but this was qualified by an interaction with the two experimental conditions, *b* = −0.498, *SE* = 0.19, 95% CI [−0.849, −0.146], *t*(288) = −2.617, *p* = 0.009, *r* = −0.152, which is displayed in Figure S4. Among participants who were competing with each other while also talking, sympathetic reactivity predicted greater friendship interest, *adj. p* < .001. Among the competing participants who showed relatively high sympathetic reactivity, talking during social interaction predicted greater friendship interest, *adj. p* = 0.017. That being said, even participants who were not that sympathetically reactive in the talking condition still had more interest in friendship when they were cooperating with their partner compared to when they were competing with their partner, *adj. p* = 0.014.


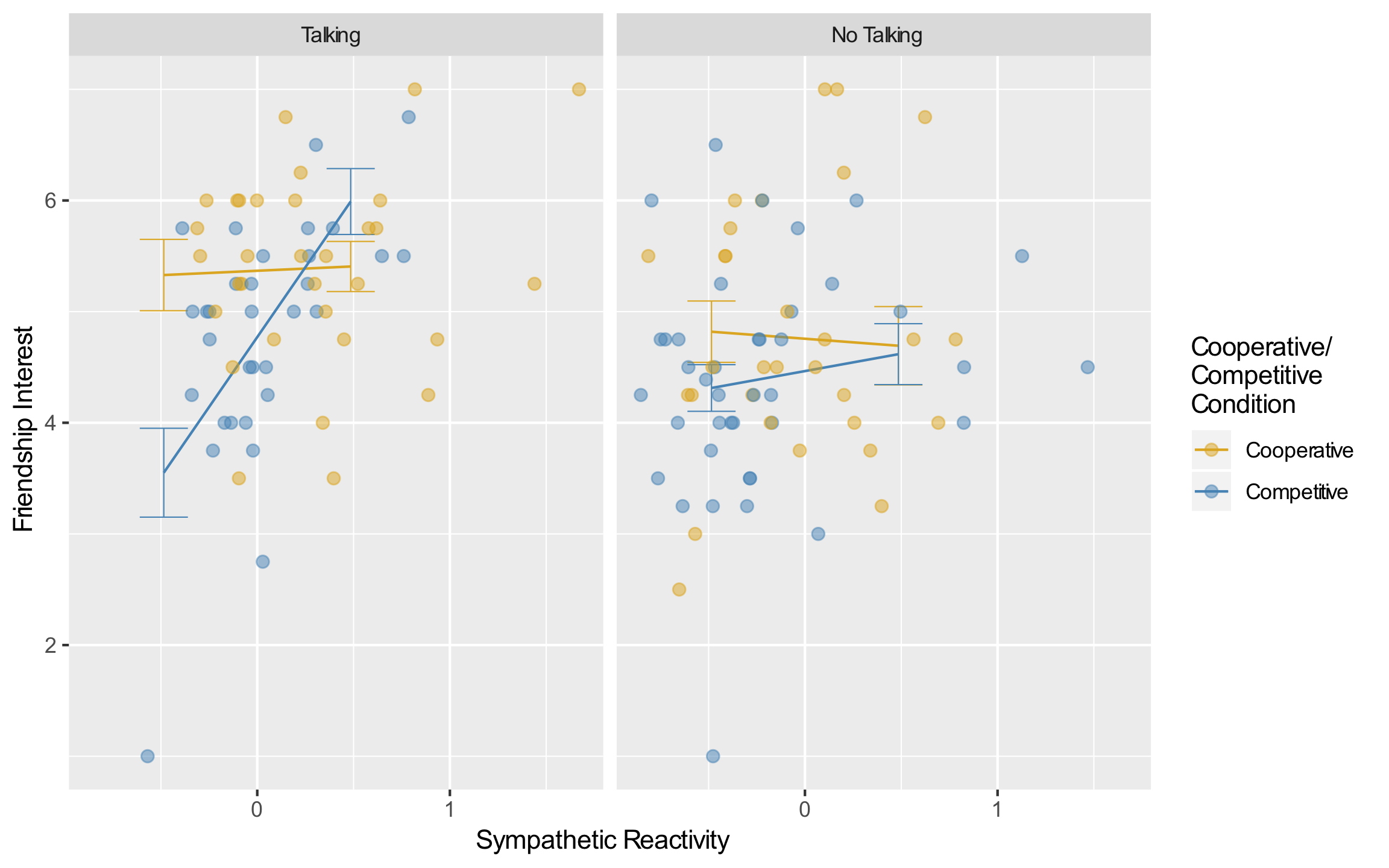


### **Figure S4.** 3-way interaction between talking conditions, cooperative/competitive conditions, and sympathetic reactivity predicting friendship interest.

***Parasympathetic Nervous System.*** In addition to the effect resported in the main text, there was also a 3-way interaction between talking/no talking, cooperative/competitive conditions, and parasympathetic reactivity, *b* = −0.402, *SE* = 0.133, 95% CI [−0.648, −0.156], *t*(288) = −3.02, *p* = 0.003, *r* = −0.175. Among participants who were assigned to compete with each other, those who showed increases in parasympathetic activity were more interested in friendship if they were talking with their partner than if they were not talking to their partner, *adj. p* = 0.045 (Figure S5).


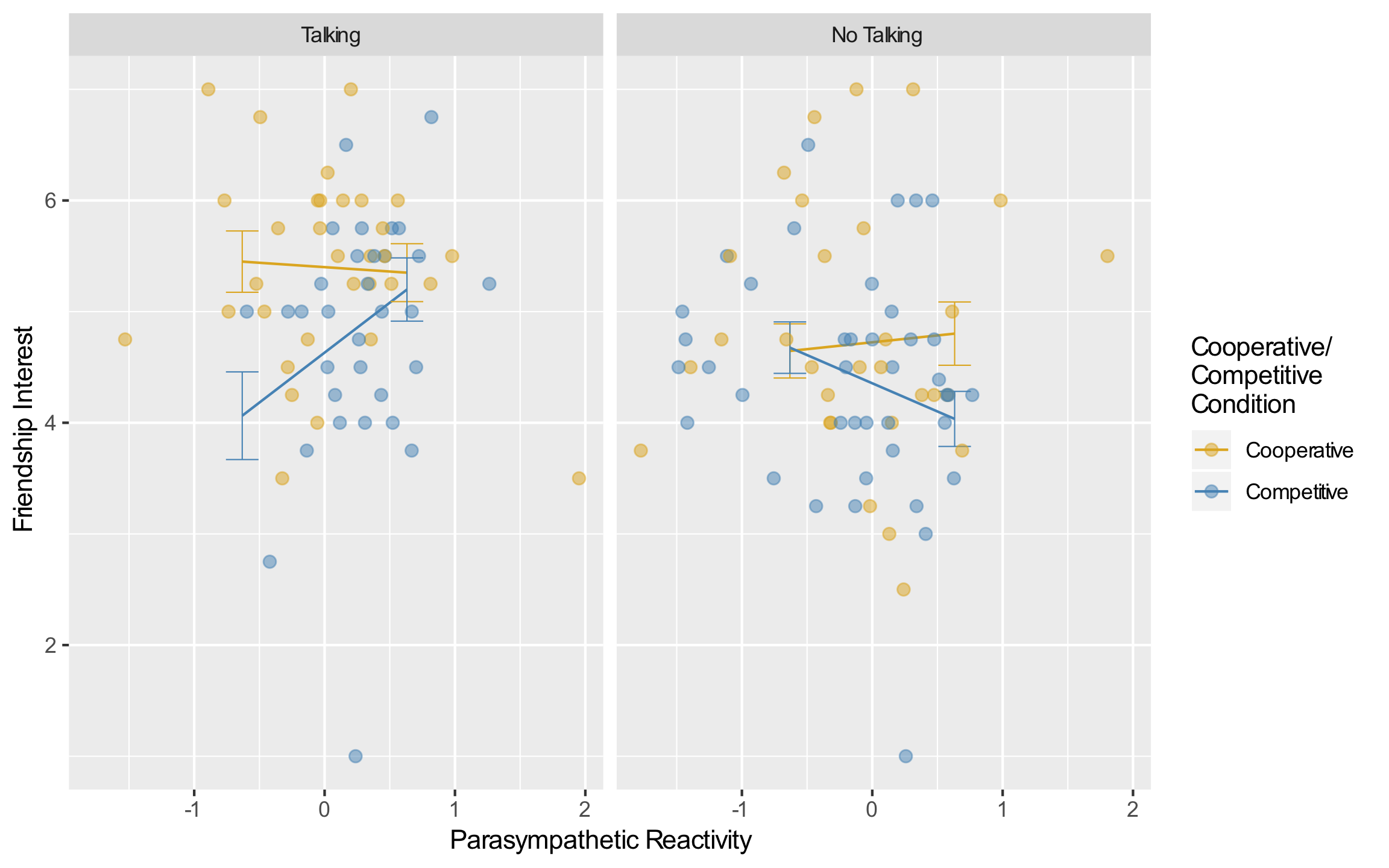


### **Figure S5.** 3-way interaction between talking conditions, cooperative/competitive conditions, and parasympathetic reactivity predicting friendship interest.

| Table S1. |  |  |  |  |  |
| --- | --- | --- | --- | --- | --- |
| *Changes in Autonomic Nervous System Activity from Baseline by Condition* | | | | | |
|  | Sympathetic Nervous System | | | | |
| Experimental Condition | *Est. Marginal Mean* | *SE* | *df* | *CI_lower_* | *CI_upper_* |
| No Talking Competitive | −0.287 | 0.114 | 63 | −0.514 | −0.0596 |
| Talking Competitive | 0.0445 | 0.127 | 63 | −0.21 | 0.299 |
| No Talking Cooperative | −0.0819 | 0.131 | 63 | −0.344 | 0.181 |
| Talking Cooperative | 0.391 | 0.127 | 63 | 0.137 | 0.645 |
|  | Parasympathetic Nervous System | | | | |
| No Talking Competitive | −0.197 | 0.119 | 63 | −0.436 | 0.0413 |
| Talking Competitive | 0.345 | 0.134 | 63 | 0.0783 | 0.612 |
| No Talking Cooperative | −0.132 | 0.138 | 63 | −0.407 | 0.144 |
| Talking Cooperative | 0.025 | 0.134 | 63 | −0.242 | 0.292 |
| *Note.* Confidence intervals that include zero indicate that the estimated marginal means do not differ from baseline*.* | | | | | |

| Table S2 |  |  |  |  |  |  |  |  |  |
| --- | --- | --- | --- | --- | --- | --- | --- | --- | --- |
| *Means, Standard Deviations, and Correlations among Measures of Physiological Synchrony, Reactivity, and Affiliation* | | | | | | | | | |
|  | a | b | c | d | e | f | g | h | i |
| a. Sympathetic Reactivity | 1 |  |  |  |  |  |  |  |  |
| b. Sympathetic Synchrony | 0.122 | 1 |  |  |  |  |  |  |  |
| c. Parasympathetic Reactivity | −0.340*** | −0.06 | 1 |  |  |  |  |  |  |
| d. Parasympathetic Synchrony | 0.018 | 0.235** | −0.223** | 1 |  |  |  |  |  |
| e. Perceived Similarity | 0.273** | 0.195* | −0.116 | −0.005 | 1 |  |  |  |  |
| f. Friendship Interest | 0.320*** | 0.021 | −0.008 | −0.041 | 0.641*** | 1 |  |  |  |
| g. Positive Affect | 0.121 | −0.095 | 0.090 | −0.167 | 0.254** | 0.330*** | 1 |  |  |
| h. Negative Affect | −0.102 | 0.022 | 0.189* | −0.025 | −0.018 | −0.105 | −0.021 | 1 |  |
| i. Email Exchange | −0.003 | 0.041 | −0.023 | 0.005 | 0.253** | 0.402*** | 0.277** | −0.091 | 1 |
| *Mean* | −0.001 | 0.000 | −0.001 | 0.000 | 4.425 | 4.824 | 0.000 | 0.000 | 0.589 |
| *SD* | 0.485 | 0.117 | 0.632 | 0.100 | 0.958 | 1.077 | 0.599 | 0.339 | 0.494 |
| *Note.* Positive and Negative Affect reflect change in affect from before to after the interaction. * *p*  ≤ .05, ** *p* < .01, *** p < .001 | | | | | | | | | |

| Table S3 |  |  |  |  |  |  |  |
| --- | --- | --- | --- | --- | --- | --- | --- |
| *Comparison of Models Predicting Sympathetic Synchrony* | | | | | | | |
| Slope for laggedpartner_jkl_ | Random Slopes at Individual Level | Covariance Structure | *df* | *AIC* | Relative Likelihood | Akaike Weight | Confidence Set |
| 1 | 1 | Unstructured |  |  |  |  |  |
| 0 | 1 | Unstructured | 16 | 2897.024 | 0.000 | 0.000 | 0 |
| 1 | 0 | Unstructured | 17 | 2898.643 | 0.000 | 0.000 | 0 |
| 0 | 0 | Unstructured | 14 | 2893.024 | 0.000 | 0.000 | 0 |
| 1 | 1 | Autoregressive | 23 | 2842.221 | 0.004 | 0.002 | 0 |
| 0 | 1 | Autoregressive | 17 | 2835.267 | 0.135 | 0.077 | 1 |
| 1 | 0 | Autoregressive | 18 | 2832.236 | 0.613 | 0.350 | 1 |
| 0 | 0 | Autoregressive | 15 | 2831.256 | 1.000 | 0.571 | 1 |

*Note*: The first and most complex model has no output, because the model failed to converge.

| Table S4 |  |  |  |  |  |  |  |
| --- | --- | --- | --- | --- | --- | --- | --- |
| *Comparison of Models Predicting Paraympathetic Synchrony* | | | | | | | |
| Slope for laggedpartner_jkl_ | Random Slopes at Individual Level | Covariance Structure | *df* | *AIC* | Relative Likelihood | Akaike Weight | Confidence Set |
| 1 | 1 | Unstructured | 22 | 2416.333 | 0.000 | 0.000 | 0 |
| 0 | 1 | Unstructured | 16 | 2428.338 | 0.000 | 0.000 | 0 |
| 1 | 0 | Unstructured | 17 | 2416.171 | 0.000 | 0.000 | 0 |
| 0 | 0 | Unstructured | 14 | 2426.504 | 0.000 | 0.000 | 0 |
| 1 | 1 | Autoregressive | 23 | 2361.616 | 0.586 | 0.255 | 1 |
| 0 | 1 | Autoregressive | 17 | 2365.481 | 0.085 | 0.037 | 0 |
| 1 | 0 | Autoregressive | 18 | 2360.546 | 1.000 | 0.436 | 1 |
| 0 | 0 | Autoregressive | 15 | 2361.488 | 0.624 | 0.272 | 1 |

| Table S5 |  |  |  |  |  |  |  |  |
| --- | --- | --- | --- | --- | --- | --- | --- | --- |
| *Model Predicting Baseline Physiological Activity by Experimental Condition* | | | | | | | | |
|  | Sympathetic Nervous System | | | | | | | |
| Term | *b* | *CI_lower_* | *CI_upper_* | *SE* | *t-value* | *df* | *p-value* | *r* |
| Intercept | 2.390 | 2.250 | 2.530 | 0.070 | 1273 | 33.720 | < .001 | 0.690 |
| Talking/No Talking | −0.150 | −0.290 | −0.010 | 0.070 | 63 | −2.070 | 0.040 | −0.250 |
| Cooperative /Competitive | 0.070 | −0.070 | 0.210 | 0.070 | 63 | 1.020 | 0.310 | 0.130 |
| Talking/No Talking × Cooperative/Competitive | −0.020 | −0.160 | 0.120 | 0.070 | 63 | −0.290 | 0.770 | −0.040 |
|  | Parasympathetic Nervous System | | | | | | | |
| Term | *b* | *CI_lower_* | *CI_upper_* | *SE* | *t-value* | *df* | *p-value* | *r* |
| Intercept | 6.030 | 5.820 | 6.230 | 0.10 | 1273 | 57.680 | 0.000 | 0.850 |
| Talking/No Talking | 0.130 | −0.080 | 0.330 | 0.10 | 63 | 1.210 | 0.230 | 0.150 |
| Cooperative /Competitive | −0.070 | −0.280 | 0.140 | 0.10 | 63 | −0.700 | 0.490 | −0.090 |
| Talking/No Talking × Cooperative/Competitive | 0.020 | −0.190 | 0.230 | 0.10 | 63 | 0.160 | 0.870 | 0.020 |
| *Note.* *b* is the unstandardized slope, *CI_lower_* and *CI_upper_* are the lower and upper bounds of the slope’s 95% confidence interval, *SE* is the standard error of the slope, *df* are the degrees of freedom for that effect, *t-value* tests whether *b* is different from zero, *p-value* reflects the probability of *t-value* given the slope is zero, and *r* is a correlation coefficient reflecting the partial effect size. The talking condition was coded with Talking = 1, No Talking = −1. The cooperative condition was coded with Cooperative = 1, Competitive = −1. Talking Cooperative (*n* = 32); Talking Competitive (*n* = 32); No Talking Cooperative (*n* = 30); No Talking Competitive (*n* = 40). | | | | | | | | |

| Table S6. |  |  |  |  |  |  |  |  |
| --- | --- | --- | --- | --- | --- | --- | --- | --- |
| *Multivariate Model Predicting Negative Affect from Physiological Synchrony and Reactivity* | | | | | | | | |
|  | Sympathetic Nervous System | | | | | | | |
| Term | *b* | *CI_lower_* | *CI_upper_* | *SE* | *t-value* | *df* | *p-value* | *r* |
| Intercept | −0.008 | −0.120 | 0.104 | 0.061 | −0.135 | 288 | 0.892 | −0.008 |
| Talking/No Talking | 0.036 | −0.076 | 0.148 | 0.061 | 0.597 | 288 | 0.551 | 0.035 |
| Cooperative/Competitive | −0.085 | −0.197 | 0.027 | 0.061 | −1.400 | 288 | 0.163 | −0.082 |
| Reactivity | −0.052 | −0.306 | 0.202 | 0.137 | −0.378 | 288 | 0.706 | −0.022 |
| Synchrony | 0.368 | −0.736 | 1.472 | 0.598 | 0.616 | 288 | 0.539 | 0.036 |
| Talking/No Talking × Cooperative/Competitive | −0.003 | −0.115 | 0.109 | 0.061 | −0.047 | 288 | 0.962 | −0.003 |
| Talking/No Talking × Reactivity | 0.062 | −0.192 | 0.316 | 0.137 | 0.450 | 288 | 0.653 | 0.027 |
| Cooperative/Competitive × Reactivity | 0.089 | −0.165 | 0.343 | 0.137 | 0.646 | 288 | 0.519 | 0.038 |
| Talking/No Talking × Synchrony | 0.193 | −0.911 | 1.297 | 0.598 | 0.323 | 288 | 0.747 | 0.019 |
| Cooperative/Competitive × Synchrony | −0.395 | −1.499 | 0.709 | 0.598 | −0.661 | 288 | 0.509 | −0.039 |
| Reactivity × Synchrony | −0.347 | −3.076 | 2.382 | 1.478 | −0.235 | 288 | 0.814 | −0.014 |
| Talking/No Talking × Cooperative/Competitive × Reactivity | −0.005 | −0.259 | 0.249 | 0.137 | −0.036 | 288 | 0.972 | −0.002 |
| Talking/No Talking × Cooperative/Competitive × Synchrony | −0.461 | −1.565 | 0.643 | 0.598 | −0.772 | 288 | 0.441 | −0.045 |
| Talking/No Talking × Reactivity × Synchrony | −1.279 | −4.008 | 1.450 | 1.478 | −0.866 | 288 | 0.387 | −0.051 |
| Cooperative/Competitive × Reactivity × Synchrony | 0.856 | −1.873 | 3.585 | 1.478 | 0.579 | 288 | 0.563 | 0.034 |
| Talking/No Talking × Cooperative/Competitive × Reactivity × Synchrony | 1.637 | −1.092 | 4.366 | 1.478 | 1.108 | 288 | 0.269 | 0.065 |
|  | Parasympathetic Nervous System | | | | | | | |
| Term | *b* | *CI_lower_* | *CI_upper_* | *SE* | *t-value* | *df* | *p-value* | *r* |
| Intercept | 0.012 | −0.097 | 0.121 | 0.059 | 0.202 | 288 | 0.840 | 0.012 |
| Talking/No Talking | −0.001 | −0.110 | 0.108 | 0.059 | −0.017 | 288 | 0.986 | −0.001 |
| Cooperative/Competitive | −0.074 | −0.183 | 0.035 | 0.059 | −1.247 | 288 | 0.213 | −0.073 |
| Reactivity | 0.124 | −0.088 | 0.336 | 0.115 | 1.077 | 288 | 0.283 | 0.063 |
| Synchrony | −0.112 | −1.273 | 1.049 | 0.629 | −0.178 | 288 | 0.859 | −0.01 |
| Talking/No Talking × Cooperative/Competitive | 0.027 | −0.082 | 0.136 | 0.059 | 0.464 | 288 | 0.643 | 0.027 |
| Talking/No Talking × Reactivity | −0.090 | −0.302 | 0.122 | 0.115 | −0.786 | 288 | 0.433 | −0.046 |
| Cooperative/Competitive × Reactivity | −0.025 | −0.237 | 0.188 | 0.115 | −0.214 | 288 | 0.831 | −0.013 |
| Talking/No Talking × Synchrony | −0.348 | −1.509 | 0.814 | 0.629 | −0.553 | 288 | 0.581 | −0.033 |
| Cooperative/Competitive × Synchrony | 0.061 | −1.100 | 1.222 | 0.629 | 0.097 | 288 | 0.923 | 0.006 |
| Reactivity × Synchrony | 0.862 | −1.607 | 3.331 | 1.337 | 0.645 | 288 | 0.520 | 0.038 |
| Talking/No Talking × Cooperative/Competitive × Reactivity | −0.039 | −0.251 | 0.173 | 0.115 | −0.337 | 288 | 0.736 | −0.02 |
| Talking/No Talking × Cooperative/Competitive × Synchrony | −0.293 | −1.454 | 0.868 | 0.629 | −0.465 | 288 | 0.642 | −0.027 |
| Talking/No Talking × Reactivity × Synchrony | 0.996 | −1.473 | 3.466 | 1.337 | 0.745 | 288 | 0.457 | 0.044 |
| Cooperative/Competitive × Reactivity × Synchrony | −0.728 | −3.197 | 1.742 | 1.337 | −0.544 | 288 | 0.587 | −0.032 |
| Talking/No Talking × Cooperative/Competitive × Reactivity × Synchrony | −1.105 | −3.574 | 1.365 | 1.337 | −0.826 | 288 | 0.409 | −0.049 |
| *Note.* *b* is the unstandardized slope, *CI_lower_* and *CI_upper_* are the lower and upper bounds of the slope’s 95% confidence interval, *SE* is the standard error of the slope, *df* are the degrees of freedom for that effect, *t-value* tests whether *b* is different from zero, *p-value* reflects the probability of *t-value* given the slope is zero, and *r* is a correlation coefficient reflecting the partial effect size. The talking condition was coded with Talking = 1, No Talking = −1. The cooperative condition was coded with Cooperative = 1, Competitive = −1. Talking Cooperative (*n* = 32); Talking Competitive (*n* = 32); No Talking Cooperative (*n* = 30); No Talking Competitive (*n* = 40). | | | | | | | | |
